# New recycled polyethylene terephthalate imidazolium ionic liquids and their applications for co_2_ exhaust capture

**DOI:** 10.1038/s41598-025-15433-7

**Published:** 2025-08-19

**Authors:** Ayman M. Atta, Alia A. Melegy, A. N. El-hoshoudy

**Affiliations:** 1 Petroleum Application Department, Egyptian Petroleum Research Institute, NasrCairo, 11727 Egypt; 2 PVT Lab, Production Department, Egyptian Petroleum Research Institute, NasrCairo, 11727 Egypt; 3 PVT Service Center, Egyptian Petroleum Research Institute, NasrCairo, 11727 Egypt

**Keywords:** Carbon dioxide (CO_2_), Recycled poly (ethylene terephthalate), Rheology, Imidazolium ionic liquids IILs, CO_2_ capture, Environmental sciences, Chemistry, Materials science

## Abstract

The recycling of polyethylene terephthalate (PET) waste using imidazolium ionic liquids IILs to capture CO_2_ exhaust offers a sustainable and environmentally friendly approach to addressing the challenges of plastic waste pollution. The functionalized imidazolium acetate with two hydroxyl and amine end groups were prepared by simple method for glycolysis and amidolysis PET to produce water soluble oligomers during short reaction time at 180 °C. ^1^HNMR analysis was used to determine the functionality and molecular weight of recycled PET oligomers with 1,3-bis(2-hydroxyethyl)-1H-imidazol-3-ium acetate ionic liquid (HIIL) and 1,3-bis(3-aminopropyl)-1H-imidazol-3-ium acetate ionic liquid (HIIL) AIIL that were 3565.7 and 1316.23 g.mol^-1^, respectively. The thermal stability and characteristics prove that the formation of elastic oligomers having higher elasticity for RPET-HIIL > RPET-AIIL that have T_g_ values for RPET-HIIL and RPET-AIIL at -69.34 and -48.44 °C, respectively with disappearance of T_g_ of PET. The CO_2_ absorption capacities at atmospheric pressure confirm that RPET-AIIL and RPET-HIIL absorb more CO_2_ with higher efficiency to about 25.2 mol CO_2_/ Kg _IIL_ and desorbs CO_2_ more easily in the short time.

## Introduction

Polyethylene terephthalate (PET) is a ubiquitous thermoplastic polymer extensively used in packaging materials, textiles, and beverage containers due to its excellent mechanical properties, transparency, and chemical resistance^1^**.** However, the widespread consumption of PET has led to significant challenges associated with plastic waste management and environmental pollution. Its extensive usage has led to environmental concerns due to its slow degradation process, contributing significantly to plastic pollution globally^2^**.** However, the proliferation of PET waste poses significant environmental challenges, necessitating the development of sustainable recycling methods. In response, there has been a growing interest in developing sustainable solutions for PET recycling and valorization. Chemical recycling of PET (using different strategies such as glycolysis, aminolysis, hydrolysis, or methanolysis) has emerged as a promising approach for the depolymerization of PET waste into valuable monomers, offering a closed-loop solution to plastic waste pollution^3^. Chemical recycling of PET waste involves the depolymerization of PET polymer chains into monomeric units through chemical reactions, typically under mild reaction conditions. Recently, ionic liquids (ILs), a class of designer solvents with unique physicochemical properties, have been investigated as efficient catalysts for PET depolymerization due to their high solvation power, tunable properties, and low environmental impact^4–7^. ILs offer several advantages over traditional solvents for PET recycling, including their ability to dissolve PET at lower temperatures and without the generation of harmful by-products. Additionally, ILs can be recycled and reused multiple times, further enhancing their sustainability credentials. Furthermore, the tunable properties of ILs allow for precise control over reaction conditions and product selectivity, enabling the optimization of PET recycling processes for maximum efficiency and yield. The recycling of PET waste using ILs aligns with broader sustainability goals and circular economy principles, emphasizing resource efficiency, waste reduction, and environmental stewardship. Moreover, ILs can be recycled and reused multiple times, further enhancing their sustainability credentials. Additionally, ILs offer tunable properties, allowing for precise control over reaction conditions and product selectivity, enabling the optimization of PET recycling processes for maximum efficiency and yield. ILs can selectively cleave ester bonds in PET molecules, leading to the recovery of monomers such as terephthalic acid (TPA) and ethylene glycol (EG), which can then be used to synthesize new PET polymers or other value-added products. There are several studies highlight the potential of ILs as versatile catalysts for PET depolymerization, enabling the valorization of PET waste into valuable chemical feedstocks. By repurposing PET waste into green and sustainable high-value chemical feedstocks, stakeholders can mitigate the environmental impact of plastic waste while creating economic value and promoting sustainable practices in the chemical and manufacturing industries. In this respect, PET was chemically recycled to produce green chemicals such as imidazolium ionic liquids (IILs) to apply for several environmental applications^8–13^. Despite the promising potential of ILs for PET recycling, challenges remain in scaling up the process to industrial levels and addressing cost considerations. Further research is needed to optimize IL formulations, develop efficient separation and purification techniques, and assess the life cycle environmental impacts of IL-based PET recycling processes.

The increasing concentration of carbon dioxide (CO₂) in the atmosphere is a major contributor to global climate change. As a result, there is a growing need for efficient and sustainable technologies to capture and sequester CO₂ from industrial flue gases and other sources. Among the various methods available, the use of ionic liquids (ILs) has emerged as a promising approach due to their unique physicochemical properties, such as low volatility, high thermal stability, tunable chemical structures, and high CO₂ solubility. ILs represent a versatile and innovative class of materials for CO₂ capture, with the potential to address many of the limitations of traditional solvents. ILs are salts in the liquid state, typically composed of organic cations and inorganic or organic anions. Their properties can be finely tuned by altering the cation–anion combination, making them highly versatile for specific applications, including CO₂ capture. There are different types of ILs used for CO₂ capture. Conventional ILs, such as those based on imidazolium, pyridinium, and ammonium cations paired with anions like [PF₆]⁻, [BF₄]⁻, or [Tf₂N]⁻, were among the first to be studied for CO₂ capture. These ILs physically absorb CO₂ through weak van der Waals interactions. For example, 1-butyl-3-methylimidazolium hexafluorophosphate ([BMIM][PF₆]) has been widely investigated for its CO₂ solubility^14^. However, conventional ILs often suffer from relatively low CO₂ absorption capacities compared to chemical absorbents like amines. Task-Specific Ionic Liquids (TSILs) are designed with functional groups that chemically interact with CO₂, enhancing absorption capacity ^**15**^. For instance, ILs with amine-functionalized cations or anions can react with CO₂ to form carbamates or carbonates. An example is 1-(3-aminopropyl)−3-methylimidazolium bis(trifluoromethylsulfonyl)imide ([APMIM][Tf₂N]), which shows significantly higher CO₂ absorption due to chemical reactions^15^. This work demonstrates the synthesis and application of TSILs for CO₂ capture, emphasizing their improved performance over conventional ILs. Protic Ionic Liquids (PILs) are formed by the transfer of a proton from a Brønsted acid to a Brønsted base. They are cost-effective and environmentally friendly, making them attractive for CO₂ capture. For example, ethyl ammonium nitrate ([EtNH₃][NO₃]) has been studied for its ability to absorb CO₂ through both physical and chemical interactions^16^. Supported Ionic Liquid Membranes (SILMs) are hybrid materials where ILs are immobilized in porous supports, such as polymeric or ceramic membranes. These systems combine the high selectivity of ILs with the mechanical stability of the support. For example, 1-ethyl-3-methylimidazolium acetate ([EMIM][Ac]) supported on a polyether sulfone membrane has shown excellent CO₂/N₂ selectivity^17^. Deep Eutectic Solvents (DESs) are mixtures of two or more components that form a eutectic with a melting point lower than that of the individual components. They are often considered analogs of ILs due to their similar properties. For example, choline chloride-urea DES has been investigated for CO₂ capture due to its low cost and biodegradability^18^. There are many challenges limited the application of ILs for CO_2_ capture^19–21^ which included high cost of some ILs limits large-scale application, viscosity can hinder mass transfer and process efficiency and limited long-term stability data for certain ILs. While challenges remain, ongoing research and pilot-scale demonstrations are paving the way for the widespread adoption of IL-based CO₂ capture technologies. For these reasons, the present work aims to prepare new functionalized imidazolium ionic liquids FIILs by condensation of glyoxal and formalin with ethanolamine (EA) or 1,3-diaminopraopane (DAP) in the presence of acetic acid as a catalyst to produce 1,3-bis(2-hydroxyethyl)−1 *H*-imidazol-3-ium acetate ionic liquid (HIIL) and 1,3-bis(3-aminopropyl)−1 *H*-imidazol-3-ium acetate ionic liquid (AIIL). Both HIIL and AIIL were used to depolymerize PET waste through transesterification or amidolysis reactions, respectively to produce water soluble oligomers functionalized with HIIL (RPET-HIIL) and AIIL (RPET-AIIL) reactive end groups. The CO_2_ exhausts capture and desorption efficiencies for the prepared HIIL, AIIL, RPET-HIIL and RPET-AIIL were investigated to alleviate the air environmental pollution from greenhouse gases and plastic solid waste. The effect of HIIL, AIIL, RPET-HIIL and RPET-AIIL viscosities on the solubility of CO_2_ gas was evaluated to optimize their capture efficacies in their aqueous solutions. This study presents an innovative approach to simultaneously address plastic waste pollution and CO₂ emissions by chemically recycling PET into functionalized imidazolium ionic liquids (IILs) for CO₂ capture. Unlike conventional PET recycling methods, which often yield low-value products or require harsh conditions, this work demonstrates the depolymerization of PET into water-soluble oligomers (RPET-HIIL and RPET-AIIL) using hydroxyl- and amine-terminated IILs (HIIL and AIIL) under mild conditions. The synthesized IILs exhibit dual functionality as efficient PET upcycling and high-performance CO₂ capture, among the highest reported for ionic liquid-based absorbents. Furthermore, the work provides mechanistic insights into CO₂ chemisorption through carbene and carbamate pathways, elucidated via NMR and spectroscopic analyses. By integrating PET waste valorization with carbon capture, this strategy offers a sustainable alternative to conventional CO₂ scrubbing technologies while contributing to the circular economy. To our knowledge, this is the first report on PET-derived IILs designed specifically for CO₂ capture, bridging the gap between plastic upcycling and greenhouse gas mitigation. The findings open new avenues for waste-to-resource conversion, though further comparative studies with existing absorbents and detailed life-cycle assessments would strengthen the practical implications of this approach.

## Experimental

### Materials

Polyethylene terephthalate (PET) was collected from utilized local Egyptian drinking bottles, converted into small pieces, cleaned with distilled water followed by acetone, and finally kept in an oven at 75 °C for drying till it attained a constant weight. The weight average molecular weight of PET was determined by gel permeation chromatography (GPC) to be close to 65,000 g mol^−1^.All chemicals were sourced from Aldrich Sigma Chemicals Co. and utilized without additional purification, with a purity level exceeding 99%. The materials included ethanolamine (EA), 1.3-diaminopropane (DAP), glyoxal monohydrate (40% GA), formalin (37% stabilized with 10–15% methanol to prevent polymerization FA), and glacial acetic acid, which were employed to synthesize functionalized ionic liquids (IILs). Additionally, saline water (5 wt.% NaCl) and deionized water were used in the experiments.

### Preparation techniques

#### Synthesis of HIIL

Ethanolamine (EA, 0.1 mol) was dissolved in 50 mL of a 50% aqueous acetic acid solution at −4°C. Separately, an aldehyde solution was prepared by dissolving glyoxal (GA, 0.05 mol) and formalin (FA, 0.05 mol) in 50 mL of 50% aqueous acetic acid at the same temperature. The EA solution was then introduced into the aldehyde solution under vigorous stirring. Reactions were conducted under a continuous N₂ purge at a flow rate of 50 mL/min for 30 min prior to heating to ensure complete oxygen removal. During the reaction, a positive pressure of N₂ (0.5 bar) was maintained to prevent air ingress. The reaction temperature was gradually raised to 70 °C and maintained for 5 h. After cooling, the mixture was washed multiple times with diethyl ether, and the solvent was evaporated under reduced pressure to yield a light yellow oil with a reaction yield of 98.3 wt.%. The product 1,3-bis(2-hydroxyethyl)−1H-imidazol-3-ium acetate ionic liquid (HIIL) was highly miscible with water (> 500 g/L) and miscible in polar solvents (methanol, ethanol, DMSO) but poorly miscible in non-polar solvents (hexane, toluene).

#### Synthesis of AIIL

An aldehyde solution was prepared by dissolving glyoxal monohydrate (5 mmol, 0.38 g) and formalin (0.005 mol) in 20 mL of aqueous acetic acid. This solution was then added to an amine solution consisting of 1,3-diaminopropane (DAP, 0.01 mol) dissolved in 20 mL of aqueous acetic acid, with the pH adjusted to 5. The reaction mixture was vigorously stirred and heated at 60 °C for 5 h. The resulting product was washed several times with diethyl ether to obtain 1,3-bis(3-aminopropyl)−1H-imidazol-3-ium acetate ionic liquid (AIIL) a viscous pale brown semisolid with a reaction yield of 95.5 wt.%. AIIL solubility in acidic, neutral and alkaline aqueous solution at pH 2, 7 and 9 water are 500, 400 and 200 g.L^−1^, respectively. AIIL was moderate soluble in alcohols, DMF; limited in chloroform/ether.

#### Recycling of PET with HIIL and AIIL

PET flakes (average size: 2–5 mm) were dried at 80 °C under vacuum (10 mbar) for 12 h before use to remove residual moisture. The glycolysis and amidolysis of PET waste were carried out by combining PET, HIIL or AIIL in a weight ratio of 1:4, respectively. The mixture was heated to 180 °C for 45 min under a nitrogen atmosphere. Ethylene glycol, a byproduct of the reaction, was separated using a Dean-Stark trap during the process. The recycled PET solutions either in HIIL or AIIL were purified using rotary evaporator under vacuum of 10 mm.Hg by separation the volatile under 50 °C for 30 min to obtain RPET-HIIL and RPET-AIIL which are not precipitate in water to produce water soluble PET oligomers. The resulting viscous recycled PET with HIIL and AIIL (RPET-HIIL and RPET-HIIL) were dried to obtain viscous pale brown oil and brown semisolid, respectively and subsequently analyzed for nitrogen content. RPET-HIIL and RPET-HIIL were soluble in water at pH from 6–8 and soluble in organic solvent such as DMF and DMSO.

### Characterization of synthesized ILs

As per the ASTM standard (ASTM-E1899), the standard method for measuring hydroxyl numbers (mg KOH/g) in HIIL and RPET-HIIL involves esterifying the hydroxyl groups with a solution of acetic anhydride in pyridine, followed by titrating the remaining acid reagent using a pre-standardized sodium hydroxide solution. The nitrogen content and total amine number (TAV; mg KOH/g) of the AIIL and RPET-AIIL, were analyzed using the Kjeldahl method, following the American Society for Testing and Materials (ASTM D 2074–19) guidelines. The TAV was determined through a back-titration method, where excess 0.5 M HCl was added to a known weight of AIIL or RPET-AIIL dissolved in 100 mL of neutral isopropanol, using bromocresol green as an indicator. The titration was performed with a standard KOH solution. The chemical structures of HIIL, AILL, RPET-HIIL and RPET-AIIL were confirmed using ^1^HNMR spectroscopy (Bruker AVANCE DRX-400) with d6-DMSO as the solvent. Thermal stability was assessed through thermogravimetric and differential thermogravimetric analysis (TGA-DTG; TGA-50 SHIMADZU) under a nitrogen atmosphere at a heating rate of 10°C/min. Additionally, the glass transition temperatures (T_g_) of HIIL, AILL, RPET-HIIL and RPET-AIIL were determined using differential scanning calorimetry (DSC; Shimadzu DTG-60 M) at a heating rate of 10°C/min.

### CO_2_ solubility, absorption, and desorption

The solubility and absorption of CO_2_ in of HIIL, and AILL or in a 70% brine water solution containing RPET-HIIL and RPET-AIIL were assessed using a bubbler setup at atmospheric pressure and room temperature, as illustrated in supplementary Figure S1. Prior to each experiment, CO_2_ gas (99.9% purity) was introduced from a cylinder into a pre-weighed bubbler (W₀). The HIIL, AILL, RPET-HIIL and RPET-AIIL solutions were then added, and the system was reweighed to determine the combined mass of the bubbler and IILs solution (W₁). CO_2_ gas was bubbled into the IILs solution at a controlled flow rate of 100 ± 5 mL/min using a regulator, while the solution was vigorously stirred with a magnetic stirrer. CO₂ absorption tests were performed in a stainless steel reactor (100 mL capacity) at 25 °C and 1 bar CO₂ pressure, with continuous stirring at 500 rpm for 2 h to ensure equilibrium. The bubbler, containing the IILs solution and absorbed CO_2_, was weighed (W₂) every 10 min to monitor the weight of absorbed CO_2_ over a period of 1–3 h until equilibrium was reached. The mass of CO_2_ absorbed was calculated as W₂—W₁ and converted into CO_2_ solubility (wt%) and moles of CO_2_ per mole of IILs based on the mass of the IILs used. For high-pressure experiments, the glass bubbler was replaced with a high-pressure stainless steel autoclave. CO_2_ was injected at a pressure of 55.2 bar, and its capture was estimated by monitoring the pressure decay until equilibrium was achieved.

CO_2_ desorption was conducted using a vacuum rotary evaporator at 55 °C and a vacuum of 200 mbar with nitrogen purging (20 mL/min) to remove CO₂. The desorption process was monitored by recording the weight loss of the IILs/water/CO_2_ solution every 5 min to determine the amount of CO_2_ released over time.

### Molecular dynamics (MD) and DFT simulation

#### MD simulation calculation

**Fig. 1:**
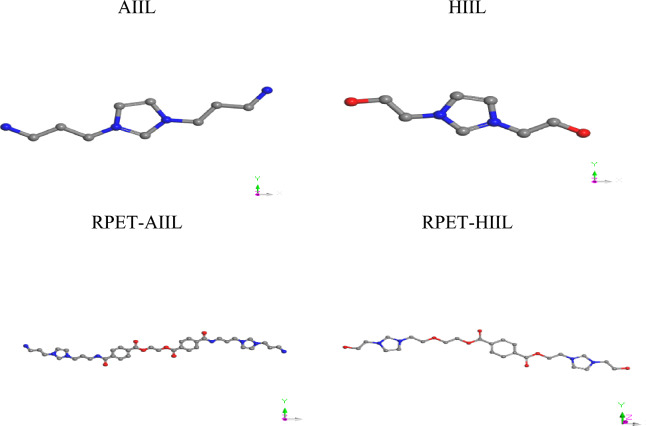
Geometrically optimized configurations of ILs.

**Fig. 2:**
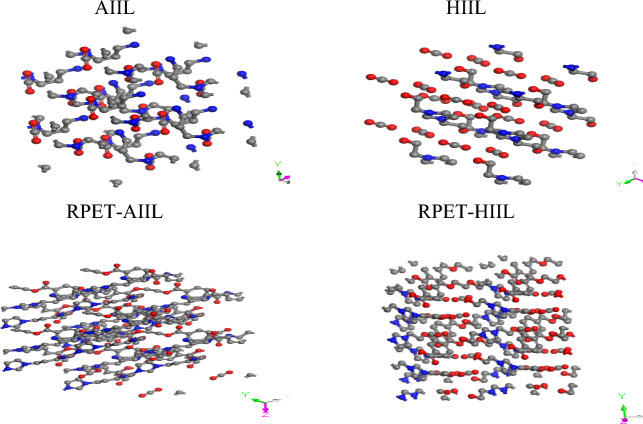
Constructed amorphous cubic cell of ILs/CO_2_.

Molecular Dynamics (MD) simulations were performed using the BIOVIA Materials Studio platform to investigate the adsorption behavior of carbon dioxide (CO₂) on various ionic liquids (ILs)^22^. Before initiating any simulations, all IL species were subjected to full geometric optimization to ensure stability and minimize internal strain (Fig. 1), as recommended by standard computational protocols^23^. The Adsorption Locator module was employed to evaluate the interaction energies between ILs and CO₂ molecules. This was achieved through the Simulated Annealing approach, set at medium computational quality^24^. The simulation was configured with three annealing cycles, each consisting of 15,000 steps, and conducted using the Smart algorithm. Energy convergence was set at 0.001 kcal/mol, while the force tolerance was maintained at 0.5 kcal/mol/Å, with a total of 500 iterations. The Universal Force Field (UFF) was applied to model atomic interactions, employing group-based electrostatics for charge distribution and atom-based van der Waals interactions to capture dispersion forces^25^. Following the adsorption energy evaluation, amorphous cells were constructed using the Amorphous Cell module. Each cell was built in a cubic lattice configuration with lattice parameters a = b = c = 2.2774 Å. Electrostatic interactions were calculated using the Ewald summation method, which provides an accurate treatment of long-range Coulombic interactions^26,27^. For each IL–CO₂ system, a molar loading ratio of 1:2 (IL: CO₂) was used to simulate realistic adsorption scenarios (Fig. 2). To further refine and validate the systems, CASTEP calculations were conducted on the generated amorphous cells. Simulations were performed at medium-quality settings, without spin polarization (non-magnetic systems), and used OTFG ultrasoft pseudopotentials for enhanced computational efficiency. The Self-Consistent Field (SCF) convergence criterion was set at 2.0 × 10⁻⁶ eV/atom, and the maximum number of SCF cycles was limited to 100, at standard electron density levels. This multi-step simulation strategy allowed for the accurate modeling of CO₂–IL interactions, providing insight into the adsorption behavior and energetic favorability of different IL candidates under idealized amorphous conditions.

#### DFT calculation

Density Functional Theory (DFT) is widely recognized as one of the most reliable computational approaches for analyzing the electronic structure and reactivity of molecular systems. In this study, DFT calculations were carried out using Spartan’14 software (version 1.1.4) to investigate the interaction mechanisms between ionic liquids (ILs) and CO₂, particularly focusing on complexation energies and chemisorption behavior relevant to gas separation performance in supported IL membranes^28^. Geometry optimization of all molecular structures was performed using the hybrid BLYP functional in combination with the 6-31G*(d,p) basis set, without imposing any structural constraints, ensuring accurate representations of equilibrium conformations. Solvation effects were included by modeling the system in water, which better simulates practical operating environments. To further probe molecular reactivity, Molecular Electrostatic Potential (MEP) maps were generated based on the calculated electron densities. MEP visualizations are crucial for identifying potential nucleophilic and electrophilic sites, which guide predictions about interaction strength and preferred binding orientations with CO₂^29^. In these maps, red-shaded regions typically represent electron-rich (nucleophilic) areas favorable for CO₂ attack, while blue regions indicate electron-deficient (electrophilic) zones. The analysis of MEP distributions thus provides valuable insights into the ILs’ capability to interact with and activate CO₂ molecules, supporting their proposed function in CO₂ capture through both electrostatic attraction and possible covalent bonding pathways.

## Results and discussion

**Scheme 1:**
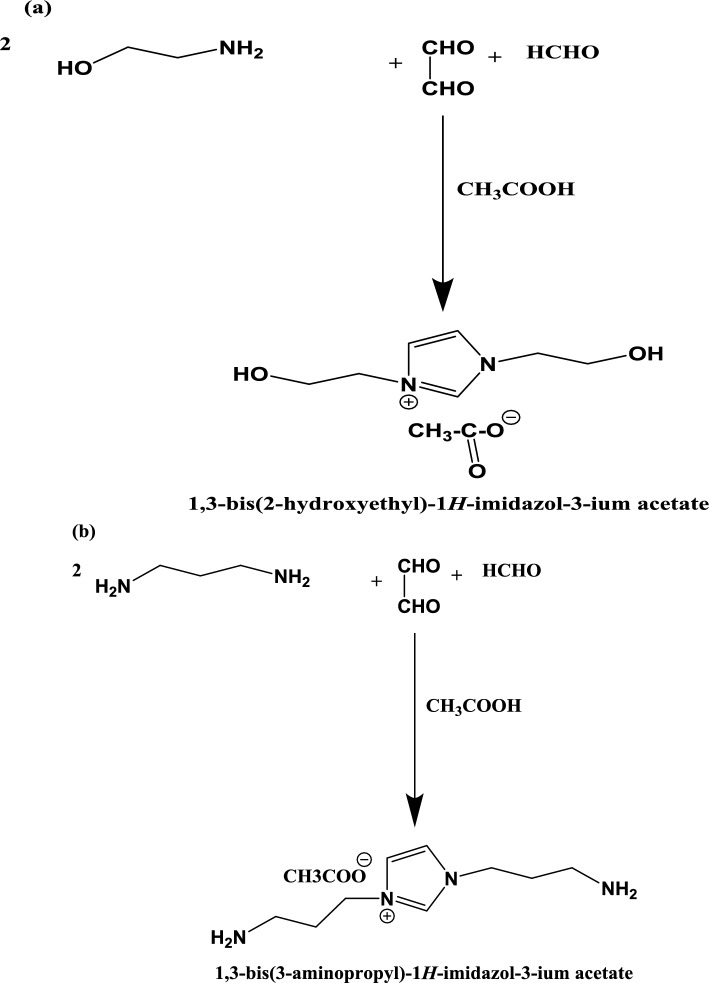
Synthesis of HIIL and AIIL.

**Scheme 2:**
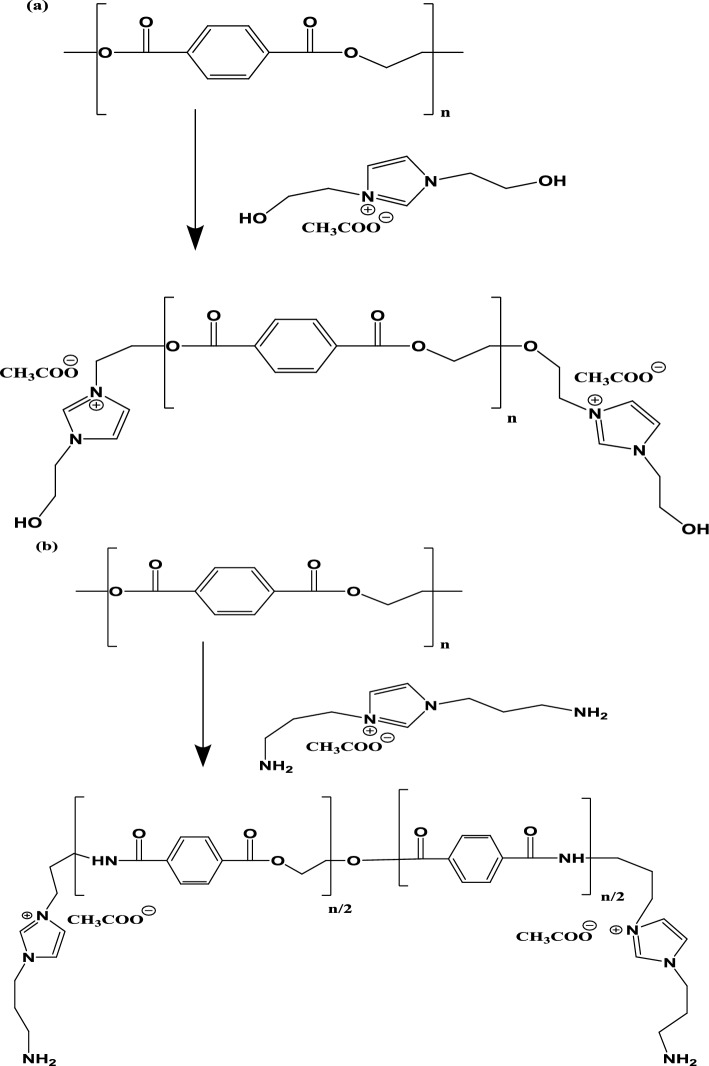
Recycling of PET with **a**) HIIL and **b**) AIIL.

The present work aims to prepare new FIILs have two ends contain hydroxyl and amino groups to solubilize the PET waste as well as to react with PET to produce new valuable products. In this respect, the preparation of bis-hydroxyethyl imidazolium acetate (HIILs) from ethanolamine, glyoxal, and formaldehyde in the presence of acetic acid is a straightforward and efficient method. The same procedure was repeated to prepare bis-aminopropylimidazolium acetate (AIILs) with replacing 1,3-diaminopropane instead of ethanolamine as reported in the experimental section. The reaction scheme was summarized in scheme 1. The reaction proceeds through cyclization and quaternization steps, yielding a functionalized ionic liquid with applications in polymer modification, catalysis, and green chemistry^22^. Characterization techniques such as ^1^H NMR, FTIR, and elemental analysis confirm the structure and purity of the product. The reaction mechanism involves the condensation reaction which EA or AP react with glyoxal and formaldehyde to form an intermediate imidazole ring. The primary amine group of EA or AP react with the aldehyde groups of glyoxal and formaldehyde, leading to cyclization and the formation of the imidazole core. The hydroxyl groups of EA and one amino groups remain intact, resulting in the formation of a bis-hydroxyethyl imidazole or bis-1,3-aminopropyl imidazole intermediate. The imidazole ring is quaternized by acetic acid, where the nitrogen atoms of the imidazole ring are protonated, and the acetate anion ([OAc]⁻) acts as the counterion. This step yields the final product, HIIL or AIIL with 98.4 and 99.6%, respectively. The HIIL and AIIL were used for chemical recycling of PET waste in absence of any catalyst at lower temperature at 170–180 °C and to reduce the reaction time to 45 min instead of 9 h through transesterification or amidation, respectively^30^. In this respect, PET: HIIL and PET: AIIL weight % 1:4 was selected to produce liquid product instead solid product that produced at 1:1 to 1:3 Wt.%. The scheme for glycolysis and aminolysis of PET through transesterification or amidation with HIIL and AIIL, respectively was represented in Scheme 2a and b. In this respect, the chemical reaction of PET with HIIL and AIIL was based on dissolution of PET by disrupting the polymer’s crystalline structure and breaking intermolecular interactions^31,32^. The acetate anion ([OAc]⁻) acts as a nucleophile, attacking the carbonyl carbon of the ester bond in PET, leading to the formation of hydroxyl and carboxylate intermediates. The imidazolium cation stabilizes the transition state and intermediates, facilitating the reaction. The hydroxyl or amine groups of HILL and AILL, respectively can react with other ester groups or intermediates as well as hydroxyl groups generated during the reaction, leading to the formation of smaller ester molecules. The HIIL or AIIL plays a dual role as a solvent and catalyst, enabling efficient transesterification and amidation of PET to produce RPET-HIIL or RPET-AIIL (Scheme 2a and b). This process has significant implications for PET recycling and the production of value-added chemicals offering a pathway for its application in sustainable chemistry and polymer science.

### Chemical and molecular structures

**Fig. 3:**
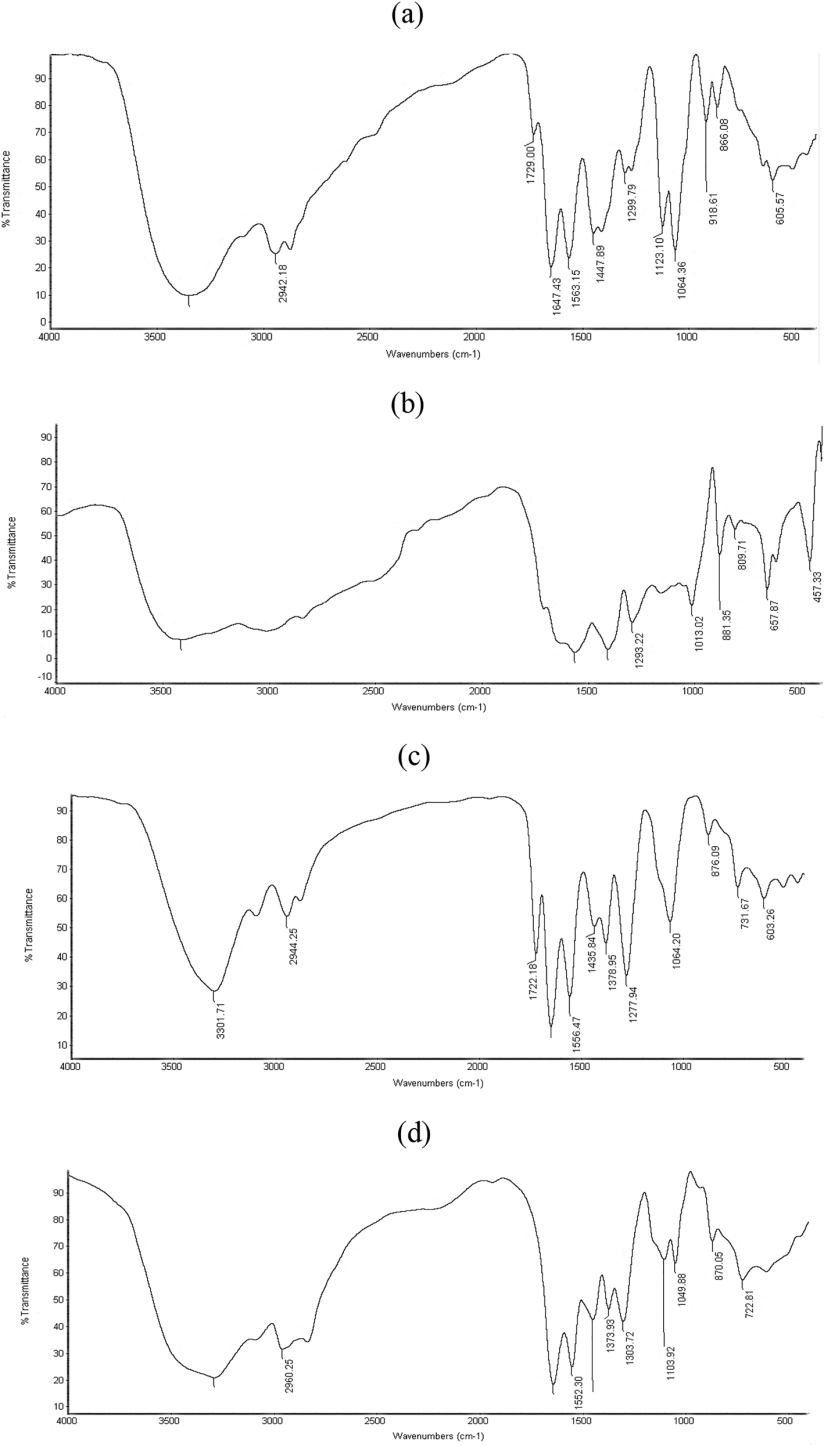
FTIR spectra of **a**) HIIL, **b**) AIIL, **c**) RPET-HIIL and **d**) RPET-AIIL.

**Fig. 4:**
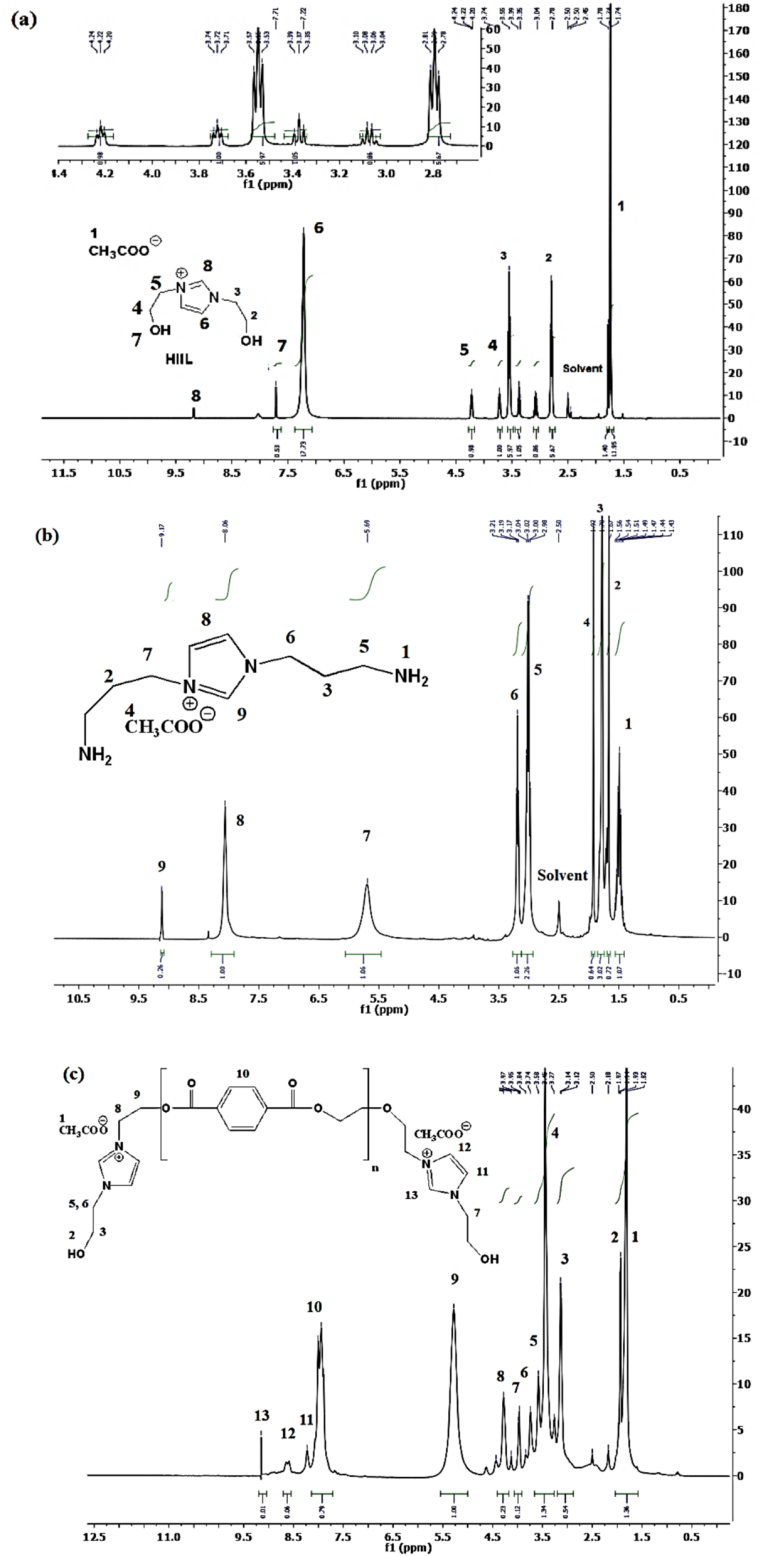

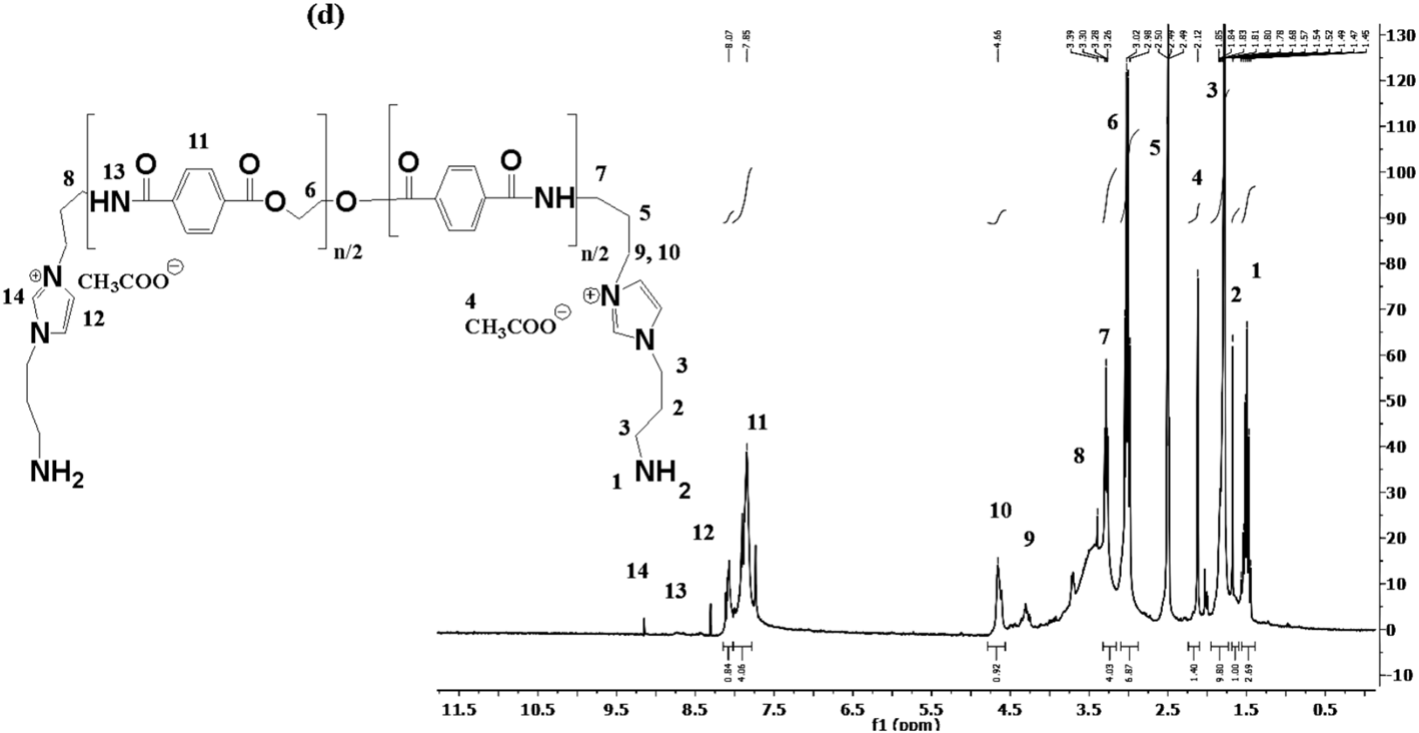
^1^HNMR spectra of **a**) HIIL, **b**) AIIL, **c**) RPET-HIIL and **d**) RPET-AIIL.

The proposed chemical structures for HIIL, AIIL, RPET-HIIL and RPET-AIIL were elucidated by FTIR and ^1^HNMR spectra summarized in Figs. 3 and 4a-d. The characteristic bands of the imidazolium cation appear in all spectra for HIIL, AIIL, RPET-HIIL and RPET-AIIL (Fig. 3a-d) in the range of 3100–3200 cm⁻^1^, corresponding to C-H stretching vibrations of the aromatic ring H-C =. Additionally, bands around 1550–1650 cm⁻^1^ are attributed to C = C and C = N stretching vibrations within the imidazolium ring. The presence of the hydroxyethyl group in spectra of HIIL and RPET-HIIL (Fig. 3a and c) is confirmed by a broad O–H stretching band around 3200–3600 cm⁻^1^. The broad O–H stretching band (3200–3600 cm⁻^1^) appeared in RPET-HIIL spectrum (Fig. 3c) shows an increase in intensity due to the generation of hydroxyl groups during the cleavage of PET ester bonds. The C-O stretching vibration of the hydroxyethyl group appears near 1050–1100 cm⁻^1^. The presence of the amino propyl groups in spectra of AIIL and RPET-AIIL (Fig. 3b and d) is also confirmed by N–H stretching vibrations around 3300–3500 cm⁻^1^ and N–H bending vibrations near 1600 cm⁻^1^. The C-N stretching vibrations appear in the range of 1000–1200 cm⁻^1^. The acetate anion ([OAc]⁻) exhibits characteristic band around 1550–1600 cm⁻^1^ (asymmetric COO⁻ stretching) and 1400–1450 cm⁻^1^ (symmetric COO⁻ stretching). A band near 1700 cm⁻^1^ may also appear, corresponding to the C = O stretching of the carboxylate group. These peaks confirm the presence of both the imidazolium cation and acetate anion in the ionic liquid, providing a baseline for understanding its interactions with PET. The bands corresponding to the imidazolium cation and acetate anion remain largely unchanged, confirming that the ionic liquid acts as a catalyst or solvent without undergoing significant chemical alteration. A strong band around 1700–1750 cm⁻^1^ corresponds to the ester carbonyl group in PET was appeared in RPET-HIIL spectrum (Fig. 3c). The transesterification of PET with HIIL involves the cleavage of ester bonds in PET and the formation of new ester linkages. As transesterification proceeds, the intensity of the ester carbonyl band (1700–1750 cm⁻^1^) in PET decreases, indicating the breakdown of ester bonds. The disappearance of PET ester bands and the appearance of amide peaks at 1690 cm⁻^1^ in RPET-AIIL spectrum (Fig. 3d) confirms the breakdown of PET and the formation of amide linkages. It was also noticed that bands around 1100–1300 cm⁻^1^ appeared in spectra of RPET-HIIL and RPET-AIIL correspond to the C-O stretching vibrations of the ester linkage of PET is essential for monitoring changes in the PET structure during transesterification and amidation reaction to confirm the proposed structures in Scheme 2a and b^24,33^..

^1^H NMR spectra of HIIL, AIIL, RPET-HIIL and RPET-AIIL (Fig. 4a-d) provide valuable insights into the structural and molecular changes during the transesterification or amidation of PET with HIIL or AILL. In this respect, all ^1^H NMR spectra (Fig. 4a-d**)** show peaks corresponding to the imidazolium ring protons C2, C4 and C5 (7.0–9.0 ppm), and acetate anion protons (1.8–2.0 ppm). The C2 proton (between the two nitrogen atoms) is usually the most deshielded and appears downfield (~ 9.0 ppm), while the C4 and C5 protons appear upfield (~ 7.0–8.0 ppm). The protons of the acetate anion ([OAc]⁻) remain largely unchanged, confirming that the HIIL and AIIL act as a catalyst or reactive solvent without undergoing significant chemical alteration during transesterification or amidation of PET. The protons of the hydroxyethyl groups (–CH₂–CH₂–OH) of HIIL (Fig. 4a) and RPET-HIIL (Fig. 4c) appear in the range of 3.5–4.5 ppm. The methylene protons adjacent to the imidazolium ring (–CH₂–N⁺) appear around 4.0–4.5 ppm, while the methylene protons adjacent to the hydroxyl group (–CH₂–OH) appear around 3.5–4.0 ppm. The hydroxyl protons (–OH) appear as a broad singlet around 4.5–5.5 ppm. The protons of the amino propyl groups (–CH₂–CH₂–CH₂–NH₂) of AIIL (Fig. 4b) and RPET-AIIL (Fig. 4d) appear in the range of 1.5–3.5 ppm. The methylene protons adjacent to the imidazolium ring (–CH₂–N⁺) appear around 3.0–3.5 ppm, while the methylene protons adjacent to the amine group (–CH₂–NH₂) appear around 2.5–3.0 ppm. The central methylene protons (–CH₂–) appear around 1.5–2.0 ppm. The appearance of PET aromatic protons of the terephthalate unit as a singlet around 8.0–8.5 ppm due to their symmetry and the methylene protons (–CH₂–) of the ethylene glycol unit as a singlet around 4.5–4.8 ppm (Fig. 4c and d) is essential for monitoring changes in the PET structure during PET transesterification and amidation reactions. As PET amidation proceeds with AILL, the intensity of the aromatic proton peak (8.0–8.5 ppm) and the ethylene glycol proton peak (4.5–4.8 ppm) decreases, indicating the breakdown of PET (Fig. 4d). New peaks corresponding to the formation of amide bonds (Fig. 4d) appear in the range of 7.5–8.5 ppm (aromatic protons adjacent to amide groups) and 2.5–3.5 ppm (methylene protons adjacent to amide groups). These peaks confirm the formation of amide linkages between the amino propyl groups of AIIL and the PET backbone^34^. The protons of the amino propyl groups (–CH₂–NH₂) (Fig. 4b and d) may shift slightly due to the formation of amide bonds. The methylene protons adjacent to the amine group (–CH₂–NH₂) may appear downfield (~ 3.0–3.5 ppm) after amidation. It was also noticed that in ^1^HNMR spectra of HIIL and RPET-HIIL (Fig. 4a and c) as transesterification proceeds, the intensity of the aromatic proton peak (8.0–8.5 ppm) and the ethylene glycol proton peak (4.5–4.8 ppm) decreases, indicating the breakdown of PET. New peaks corresponding to the formation of smaller ester molecules may appear in the range of 3.5–4.5 ppm (methylene protons adjacent to new ester groups) and 8.0–8.5 ppm (aromatic protons adjacent to new ester groups). These peaks confirm the formation of new ester linkages. The protons of the hydroxyethyl groups (–CH₂–OH) may shift slightly due to the formation of new ester bonds. The hydroxyl protons (–OH) may appear as a broad singlet around 4.5–5.5 ppm.

It was previously reported that^35–37^, the ^1^HNMR is an effective tool to determine the molecular weight of RPET waste through transesterification or aminolysis and amidation reactions that also confirmed by hydroxyl (HV; mgKOH.g^−1^ polymer) and amine values (AV; mgKOH.g^−1^ polymer). The number average molecular weight (Mn; g.mol^−1^) of RPET-HIIL and RPET-AIIL was determined by calculation the degree of polymerization (DP) from equation; DP = [(integration of repeating unit/number of protons) (number of end group protons/integration of end group protons) followed by multiplication in molecular weight of repeating unit. The Mn, HV and AV of RPET-HIIL and RPET-AIIL were determined and listed in Table1. The lowering of Mn value for RPET-AIIL than RPET-HIIL confirms that the amidation was more effective to produce lower molecular weights recycled PET oligomers than transesterification.

**Table 1:** Physico-chemical characteristics of the prepared IILs.

IIL Types	Hydroxyl value mgKOH.g^−1^	Amine value mgKOH.g^−1^	Mn g.mol^−1^	DP	Density g.cm^−3^	Refractive index	Kinematic viscosity cP
HIIL	518.65 ± 0.35	-	-	-	1.186 ± 0.004	1.482 ± 0.001	200 ± 0.03
AIIL	-	928.83 ± 0.15	-	-	-	-	80 ± 0.08*
RPET-HIIL	206.23 ± 0.24	-	3565.7	19	1.216 ± 0.003	1.554 ± 0.002	350 ± 0.01
RPET-AIIL	-	564.68 ± 0.13	1316.23	8	-	-	120 ± 0.04*

* measured in aqueous solution (80 Wt. %).

The solubility of the produced oligomers from PET recycling in water was tested to confirm the functionality of the produced oligomers. In this respect, the term water-soluble oligomers refer to the practical solubility of RPET-HIIL and RPET-AIIL under the tested conditions of 10–50 mg/mL in deionized water (25°C) with mild stirring (200 rpm, 1 h). RPET-HIIL (pH 6–8) was completely soluble due to hydroxyl-terminated groups enhancing hydrophilicity. RPET-AIIL (pH 7–9) has solubility > 90%, aided by amine groups that protonate under acidic conditions (pH < 6). The DP values in Table 1 are now contextualized with the initial PET feedstock (DP ~ 300–320, Mₙ ~ 60,000 g/mol). DP of RPET-HIIL reduced from 300 to 19 (Mₙ = 3565.7 g/mol), achieving 90% chain scission. DP of RPET-AIIL reduced to 8 (Mₙ = 1316.23 g/mol), achieving 95% chain scission. AIIL’s lower DP aligns with its amine-mediated nucleophilic attack, which cleaves ester bonds more aggressively than HIIL’s hydroxyl-driven hydrolysis. The residual oligomers (DP > 2) are intentional for functionalization retaining short chains (DP 2–10) ensures water solubility while preserving terephthalate/imidazolium motifs for CO₂ capture. Conventional glycolysis typically yields DP 1–3; our method balances solubility (higher DP) with functionality.

### Thermal stability and characteristics

**Fig. 5:**
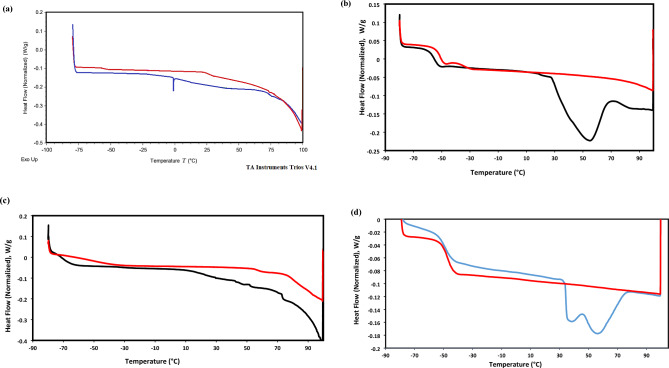
DSC thermograms of **a**) HIIL, **b**) AIIL, **c**) RPET-HIIL and **d**) RPET-AIIL.

DSC thermograms of ILs provide critical insights into its thermal behavior and phase transitions, including glass transition (T_g_), melting (T_m_), and crystallization (T_c_) temperatures. DSC analysis is essential for understanding the thermal properties of ILs and optimizing its applications in green chemistry, and energy storage. In this respect, DSC thermograms of HIIL, AIIL, RPET-HIIL and RPET-AIIL were represented in Fig. 5a-d. It was noticed that HIIL, AIIL, RPET-HIIL and RPET-AIIL have not Tc data to confirm their semi-crystalline and amorphous nature^38^. The prepared HIIL, and RPET-HIIL are viscous at room temperature and their DSC thermograms confirm the absence of T_m_ data even when heated or cooled several cycles. AIIL and RPET-AIIL have T_m_ endothermic peaks at 53.43 °C (Enthalpy 38.61 J.g^−1^) and two peaks at 35.72 °C (enthalpy 3.38 J.g^−1^) 57.19 °C (enthalpy 7.74 J.g^−1^), respectively. The Two double endothermic peaks with low enthalpy values of RPET-AIIL were referred to melting of the original crystal and the melting of crystal formed during the melt and recrystallization process during a heating scan, respectively. The same character was reported and elucidated by X-ray diffraction for different types of IILs^38,39^**.** The AIIL, RPET-HIIL and RPET-AIIL (Fig. 5b-d) show lower T_g_ values recorded at −55.16, −69.34 and −48.44 °C, respectively. These data elucidate that the elasticity of the oligomer chains of the prepared recycled PET-ILs is arranged in the order RPET-HIIL > RPET-AIIL. It is well known that the T_g_ value was influenced by the flexibility of the hydroxyethyl side chains and the ionic interactions within the IL. The presence of hydrogen bonding between the hydroxyl groups of RPET-HIIL and the acetate anion may also affect the T_g_^40^. It is well known that, the T_g_ of PET is typically observed in the range of 70 °C to 80 °C. The lowering of T_g_ values for RPET-HIIL and RPET-AIIL (Fig. 5b-d) at −69.34 and −48.44 °C, respectively and disappearance of T_g_ of PET confirm the formation of elastic oligomers having higher elasticity for RPET-HIIL > RPET-AIIL that facilitated their applications. Accordingly, it can be concluded that, the transesterification of PET with HIIL produces more flexible oligomer chains having lower molecular weights than amidation of PET with AIIL. This was referred to strong ion pairing and strong hydrogen bonding of RPET-AIIL with acetate ions to increase its T_g_ value due to increasing of cohesion and structural rigidity of oligomer chains^41^.

**Fig. 6:**
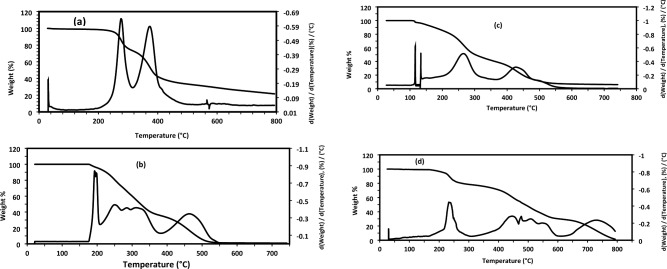
TGA-DTG thermograms of **a**) HIIL, **b**) AIIL, **c**) RPET-HIIL and **d**) RPET-AIIL.

Thermogravimetric differential thermal analysis (TGA-DTG) is a widely used technique to study the thermal stability and decomposition behavior of the prepared ILs and their oligomers with RPET waste. TGA-DTG thermograms of HIIL, AIIL, RPET-HIIL and RPET-AIIL were summarized in Fig. 6a-d to provide critical insights into their thermal degradation profile, decomposition temperature, and weight loss behavior. It is well known that, the thermal stability `of IILs is influenced by the structure of anion, cations and the presence of impurities ^**35**^. It was also reported that the acetate is a basic anion tends to lower thermal stability of IILs because acetate can undergo decomposition at elevated temperatures, leading to the formation of CO_2_ and volatile by-products ^**35**^. For example, 1-ethyl-3-methylimidazolium acetate has been reported to start decomposition around 200 °C under inert atmosphere^42^. The presence of water impurities combined with IILs can significantly reduce their thermal stability. The TGA-DTG thermograms of HIIL, AIIL, RPET-HIIL and RPET-AIIL, Fig. 6a-d, show their initial degradation temperature (IDT, ^o^C), thermal degradation steps and residual contents at 600 °C (Rs. %, Wt. %) without degradation step below 150 °C except RPET-AIIL to confirm the absence of water humidity combined with hydrophilic ILs. The IDT of HIIL, AIIL, RPET-HIIL and RPET-AIIL are 255.5, 193.5, 207.3 and 141.3 °C, respectively (Fig. 5a-d). A small weight loss may occur at lower temperatures (below 100 °C) for RPET-AIIL (Fig. 5d) due to the evaporation of absorbed moisture or residual solvents. A sharp weight loss corresponding to the breakdown of the IILs structure, including the imidazolium cation and acetate anion was stated at 295.6, 255.4, 323.2 and 257.6 °C for HIIL, AIIL, RPET-HIIL and RPET-AIIL respectively. It is well known that the sharp weight loss of PET is typically observed in the range of 350–380 °C. This temperature marks the beginning of the thermal decomposition of PET. The major weight loss occurs in the temperature range of 400–500 °C. This step corresponds to the breakdown of the ester linkages and the release of volatile compounds such as CO₂, H₂O, and small organic molecules. The incorporation of HIIL and AIIL reduces the thermal stability of PET with the formation of RPET-HIIL and RPET-AIIL. This shift is due to the introduction of ester and amide linkages as well as the presence of HIIL and AIIL, which may lower the thermal stability of the polymer due to lowering of their basicity^42^. The Rs. % of HIIL, AIIL, RPET-HIIL and RPET-AIIL are 22.5, 1.2, 26.3 and 7.5%, respectively indicating that RPET decomposes almost completely without leaving significant residues. The HIIL and AIIL have higher Rs. % to elucidate that they form crosslinked cyclic carbon and nitrogen residual due to the presence of hydroxyl and amino groups as well as acidic C2-H of imidazolium cation that can polymerize or cross-link during decomposition. It was reported that the imidazolium-based cations are relatively stable but can form stable carbonaceous residues upon decomposition^43^. The higher Rs. % in RPET-HIIL vs. HIIL arises from PET backbone integration into the char matrix. Elemental analysis (EA) of residues revealed a C/N molar ratio of 5.2 for HIIL and 4.8 for RPET-HIIL, consistent with nitrogen-doped carbonaceous chars. The negligible residue for AIIL (vs. HIIL) initially appears contradictory but is explained by volatilization of amine termini which NH₂ groups decompose as NH₃/volatile nitriles**.** Unlike HIIL’s –OH groups, which promote char formation via dehydration/aromatization, AIIL’s amines favor chain scission. It is expected that HIIL yields stable furan/pyridine derivatives, while AIIL produces volatile nitriles. While HIIL and RPET-HIIL form N-doped carbonaceous chars via hydroxyl-assisted crosslinking, AIIL’s amine groups volatilize as small molecules (NH₃, nitriles), accounting for its minimal residue.

### CO_2_ absorption desorption mechanism

**Fig. 7:**
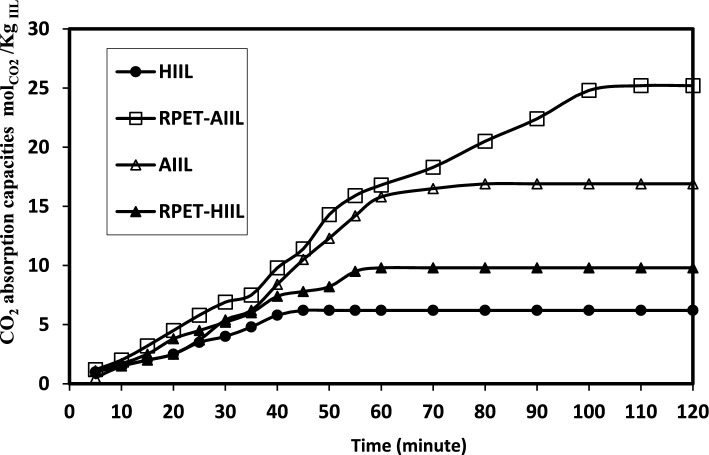
Relations of CO_2_ uptakes of the prepared IILs and time at room temperature.

**Scheme 3:**
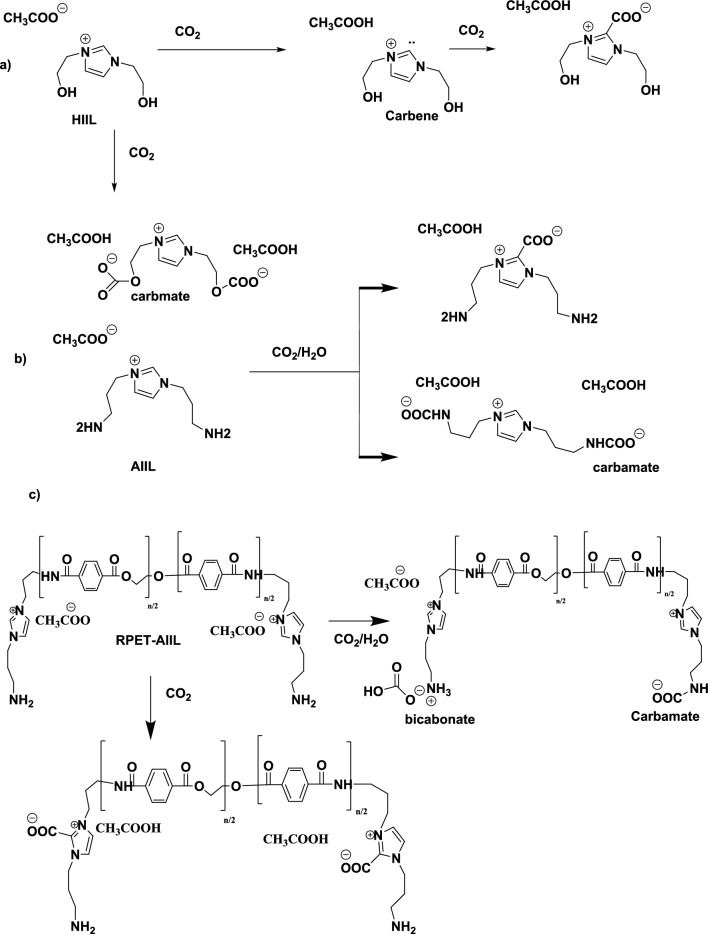
CO_2_ chemisorption mechanism of a) HIIL, b) AIIL and c) RPET-AIIL.

The solubility of CO₂ in liquids and viscous polymers such as polyester and polyamide depends on several factors, including the chemical structure of the polymer, temperature, pressure, and the presence of other additives or fillers. Polyesters, such as PET, are semi-crystalline polymers with moderate gas barrier properties. CO₂ solubility in polyesters is relatively low compared to other polymers like rubbers or elastomers. This is due to the relatively dense and polar nature of polyester chains, which limits the diffusion and absorption of non-polar gases like CO₂. The solubility of CO₂ in PET, for example, is typically in the range of 0.1–0.3 cm^3^(STP)/cm^3^·atm at room temperature and moderate pressures^44^. The present work aims to recycle PET and to increase its CO_2_ gas solubility with incorporating HIIL and AIIL. The relation between CO_2_ capture capacities (mol CO_2_/Kg IIL) of HIIL, AIIL, RPET-HIIL and RPET-AIIL versus time to attain equilibrium was represented in Fig. 7. The data of CO_2_ absorption capacities and time to attain equilibrium were summarized in Table 2. The AIIL and RPET-AIIL were mixed with distilled water (80 Wt. %) to convert to liquids due to their melting temperature above 50 °C (Fig. 5b and d). It is expected that the CO₂ solubility of RPET-AIIL is generally higher than, RPET-HIIL, AIIL and HIIL due to the polar nature of the amide groups, which can interact with CO₂ molecules^45^. The DSC curves of HIIL, AIIL, RPET-HIIL and RPET-AIIL (Fig. 5a-d) show their amorphous structure due to absence of crystallization temperature which will be more permeable to gases and increase their solubility with CO₂. The solubility of CO₂ in RPET generally higher than in unmodified PET due to increased free volume and reduced crystallinity. The addition of imidazolium ILs to RPET can significantly increase CO₂ solubility. The ILs create additional sites for CO₂ absorption through physical interactions (e.g., van der Waals forces) and potentially chemical interactions (e.g., Lewis acid–base interactions). The presence of ILs further disrupts the polymer matrix, increasing free volume and gas absorption. The proposed CO_2_ chemical sorption of HIIL, AIIL and RPET-AIIL can be represented in Scheme 3** a-c**. It is expected that the carbene mechanism was obtained at C2 of imidazolium cations which has strong probability when C-H at C-2 was more acidic and anions more basic such as acetate anion^46,47^. It was proposed that the CO_2_ absorption capacity by chemisorption of imidazolium cations using carbene mechanism is 0.5 mol/mol _IILs_. Moreover, the hydroxyl group, ether and amine functional groups were chemically bonded with CO_2_ through formation of carbamate group with absorption capacity 1.5 mol/mol_IILs_^48,49^. The chemical absorption of CO_2_ with IIL through interaction of CO_2_ with cations via carbamate complex mechanism followed by the deprotonation of cation that depends on the hydrogen bonding of the acidic hydrogen protons of the cations and anions^50^. Moreover, it is proposed that the amount of carbamate cation complex should provide an indication of the strength of hydrogen bonding between cations and anions when the prepared IIL absorb CO_2_ (as illustrated in the scheme 3a-c). The data of CO_2_ absorption capacities (Table 2** and **Fig. 7) elucidated that the prepared IIL were arranged in the order RPRT-AIIL > AIIL > RPET-HIIL > HIIL for CO_2_ capture uptake. RPET-AIIL is a polymer with amide linkages and two amines and imidazolium end groups when functionalized with AIIL, the end groups contain imidazolium cations and acetate anions provide CO₂ capture sites, while the polymer backbone offers structural stability and tunable properties. The presence of both amine and imidazolium functional groups in AIIL enables it to interact with CO₂ through chemical reactions, making it a promising candidate for CO₂ capture and utilization. The interaction of AILL with CO₂ involves a combination of physical absorption and chemical reactions. CO₂ is initially absorbed into the ionic liquid due to its high solubility AIIL. The imidazolium cation and acetate anion create a polar environment that facilitates the dissolution of CO₂. The amine groups (–NH₂) in the amino propyl side chains of AIIL react with CO₂ to form carbamate and bicarbonate species. This reaction is facilitated by the nucleophilic nature of the amine groups. The primary amine groups react with CO₂ to form carbamate ions. The carbamate ions can further react to form bicarbonate ions in the presence of water. The imidazolium cation stabilizes the carbamate and bicarbonate ions through electrostatic interactions. The acetate anion may also participate in stabilizing the reaction intermediates. Water plays a crucial role in the formation of bicarbonate ions. However, excessive water may dilute the ionic liquid and reduce its CO₂ capture efficiency. The length and flexibility of the amino propyl side chains, as well as the nature of the imidazolium cation and acetate anion, influence the reactivity and stability of the reaction intermediates. HIIL consists of an imidazolium cation with two hydroxyethyl groups (–CH₂-CH₂-OH) and an acetate anion ([OAc]⁻). The hydroxyethyl groups can interact with CO₂ through physical absorption and weak chemical interactions, while the imidazolium cation and acetate anion provide a polar environment that enhances CO₂ solubility. RPET-HIIL is a polymer with ester linkages and hydroxyl end groups when functionalized with HIIL, the end groups contain imidazolium cations and acetate anions provide CO₂ capture sites, while the polymer backbone offers structural stability and tunable properties. The ester linkages and hydroxyl groups may also participate in CO₂ interactions. CO₂ is primarily absorbed through physical interactions with the polar imidazolium cation and acetate anion. The hydroxyethyl groups may form weak hydrogen bonds with CO₂, enhancing solubility but not participating in strong chemical reactions. RPET-HIIL backbone may provide additional sites for CO₂ absorption, depending on the degree of functionalization and the presence of ester and hydroxyl groups. The CO₂ absorption capacity of HIIL is moderate, typically in the range of 0.1–0.3 mol of CO₂ per mole of IL, depending on temperature and pressure. The absorption capacity of RPET-HIIL may be higher than pure HIIL due to the additional CO₂ interaction sites provided by the polymer backbone (Fig. 7** and **Table 2). The absorption kinetics are relatively fast due to the high polarity and fluidity of the IL. However, the lack of strong chemical interactions limits the overall absorption efficiency. The absorption kinetics of RPET-HIIL may be slower compared to pure HIIL due to the semi-solid nature of the polymer. The diffusion of CO₂ into the polymer matrix may be a rate-limiting step. It was previously reported that, the amine-based solvents, such as ethanolamine (MEA) and diaminopropane (DAP), are widely used for CO₂ capture due to their ability to chemically react with CO₂^**44**^. DAP also exhibits fast reaction kinetics with CO₂, similar to MEA. The presence of two amine groups may further enhance the reaction rate. The formation of carbamate and bicarbonate species in DAP may be more complex due to the presence of two reactive sites. Both primary amine groups can react with CO₂, potentially increasing the CO₂ absorption capacity compared to MEA. MEA has a CO₂ absorption capacity of approximately 0.5–0.6 mol of CO₂ per mole of MEA. This is due to the formation of carbamate, which limits the stoichiometric ratio to 1:2 (CO₂: MEA). DAP has a higher CO₂ absorption capacity compared to MEA, typically around 1.0–1.2 mol of CO₂ per mole of DAP. This is because both amine groups can react with CO₂, potentially doubling the absorption capacity.

**Table 2:** The CO_2_ absorption capacities of the prepared IILs at equilibrium under atmospheric pressure and their desorption time under vacuum.

IILs	CO_2_ absorption at atmospheric pressure		Desorption time (min)
	absorption capacities (mol_CO2_/Kg _IIL_)	Time (min)	
HIIL	6.203 ± 0.001	45 ± 0.20	27 ± 0.14
AIIL	16.914 ± 0.002	80 ± 0.53	50 ± 0.25
RPET-HIIL	9.823 ± 0.001	60 ± 0.45	18 ± 0.13
RPET-AIIL	25.246 ± 0.005	110 ± 0.85	40 ± 0.15

**Fig. 8:**
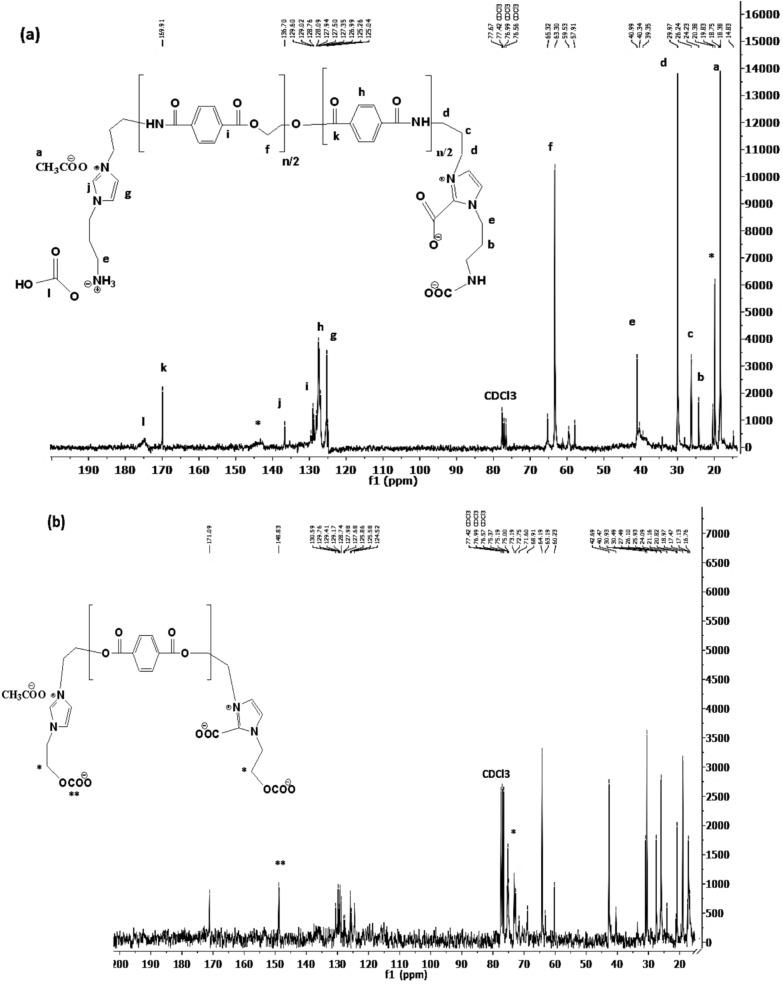
^13^CNMR spectra of **a**) RPET-AIIL and **b**) RPET-HIIL after CO_2_ absorption.

It was previously reported that the CO_2_ absorption mechanism of IILs was proved by ^1^HNMR and ^13^CNMR spectra^51^. In this respect, ^13^CNMR spectra of RPET-AIIL and RPET-HIIL were selected as representative and summarized in Figs. 8a and b, respectively. The chemical structures of RPET-AIIL (Fig. 8a) was marked with their peaks at chemical shifts to confirm the predicted chemical structures in scheme 2. The appearance of new peak ranged from 69 to 72 ppm in ^13^CNMR spectrum of RPET-HIIL (Fig. 8b) elucidates the formation of CH_2_-O-COO^-^ (scheme 3a-c) due to chemisorption of CO_2_ with hydroxyl terminated IILs as well as new peak at 148 ppm of OCOO group^51^. Both RPET-AIIL and RPET-HIIL spectra (Fig. 7a and b) show the presence of acetic acid peaks at 19–21 and 144–146 ppm which attributed to CH_3_ and COOH groups to elucidate the CO_2_ chemisorption by deprotonation of C-2 with carbine mechanism (as illustrated in Scheme 2)^52^.

**Fig. 9:**
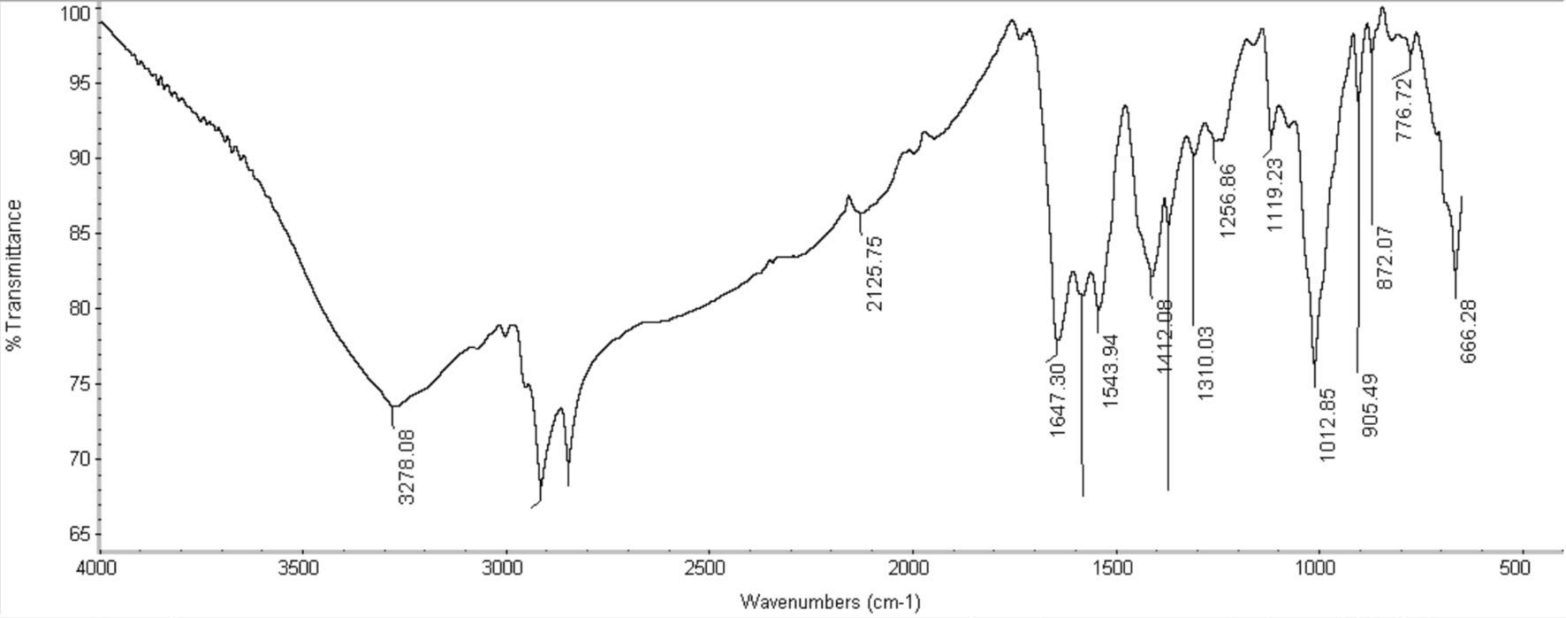
FTIR spectrum of RPET-AILL after CO_2_ absorption.

FTIR was used also to elucidate the chemical interaction of CO_2_ with the prepared IILs which RPET-AIIL was selected as representative sample in Fig. 9. FTIR of CO₂ absorption in the RPET-AIIL (Fig. 3d) revealed the gradual appearance of a new bands at wavenumber 2125 cm^−1^, and 650 −680 cm^−1^ attributed to O = C = O stretching and bending, respectively were recorded (characteristic of the C = O stretch in imidazolium-2-carboxylates carbene-CO₂ adducts; Scheme 2) supporting the proposed reaction pathway. Bicarbonate formation (which typically shows broad peaks at ~ 1647 cm⁻^1^ and ~ 1350 cm⁻^1^) was negligible under anhydrous conditions, further confirming the dominance of the carbene route.

**Fig. 10:**
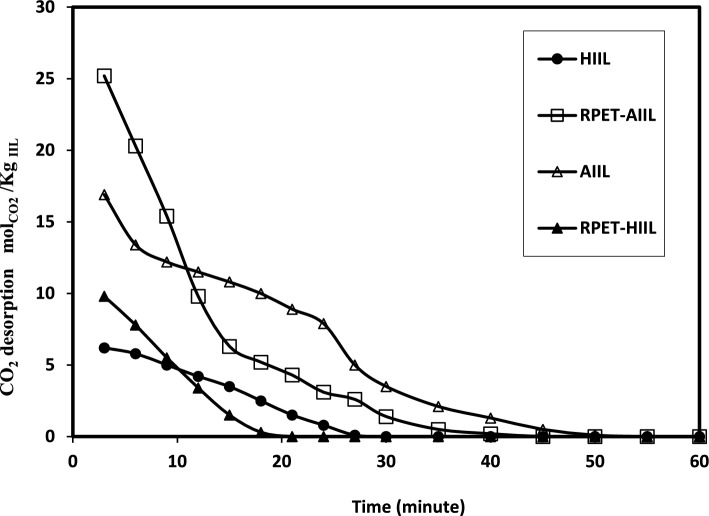
Relations of CO_2_ desorption of the prepared IILs with time under vacuum.

The CO_2_ desorption data were measured according to the procedure reported in the experimental section using rotary evaporator under reduced pressure. The desorption data of the pure IILs were represented in Fig. 10. The results **(**Fig. 10) show that the RPET- HIIL desorbs CO_2_ more easily during 15 min than HIIL, RPET-AIIL and AIIL that desorb CO_2_ during 25, 35 and 45 min, respectively. The formation of imidazolium cations at the end group facilitates both absorption desorption of CO_2_ from their solutions. The fast reaction can lead to the formation of stable carbamate, which may require higher energy for regeneration. It was reported that, the regeneration of MEA requires significant energy input, typically around 3.5–4.0 GJ/tonne of CO₂. This is due to the strong binding of CO₂ to the amine group and the need to break the carbamate bond^53^. The regeneration of DAP may require slightly less energy compared to MEA, depending on the stability of the carbamate and bicarbonate species formed. However, the energy requirements are still significant, typically around 3.0–3.5 GJ/tonne of CO₂. The activity of RPET-HIIL, HIIL, RPET-AIIL and AIIL for regeneration to the CO_2_ absorption and desorption was examined and they reused without changing in their efficiencies for 14, 12, 16 and 10 cycles, respectively. These data confirm that RPET-AIIL and RPET-HIIL absorb more CO_2_ with higher efficiency and desorbs CO_2_ more easily in the short time as represented in Fig. 10**.**

Table 3 summarized CO_2_ absorption data comparing RPET-AIIL’s CO₂ absorption performance such as CO_2_ Absorption capacity (mol/kg), viscosity, regeneration cycles (RC), and desorption time (DT) at definite desorption temperature at 55 °C and a vacuum of 200 mbar with functionalized ILs from recent literature. They are including amino-functionalized AFPILs (e.g., [P4444][Gly], [P66614][2-CNpyr]), task-specific ILs (TSILs) (e.g., [emim][Ac], [bmim][BF₄]) and hybrid solvents (e.g., MEA-[TBP][MeSO₃]). RPET-AIIL’s capacity (25.2 mol/kg) exceeds most PILs/TSILs (typically 0.5–10 mol/kg) and matches amino-acid-based ILs (e.g., [P66614][Triz] at 24.8 mol/kg). Unlike conventional ILs, RPET-AIIL (aqueous solution 80 Wt.%) combines high capacity with low viscosity (120 cP at 25 °C), addressing mass transfer limitations common in viscous TSILs. Regenerability (10–16 cycles) outperforms many TSILs (e.g., [emim][Ac] degrades after 5 cycles).

**Table 3:** CO_2_ absorption data comparing RPET-AIIL’s CO₂ absorption performance with functionalized ILs from recent literature.

ILs Type	symbol	CO_2_ Absorption capacity (mol_CO2_/Kg _IIL_)	Viscosity cP	RC (cycle)	DT (minute)	Reference
IILs	RPET-AIIL	25.2	120	16	45	This work
AFPILs	[P4444][Gly], [P66614][2-CNpyr]	24.8	400–1200	10	50–80	^54,55^
TSILs	[emim][Ac], [bmim][BF₄]	0.5–10	250–900	5	40–90	^54,56^
Hybrid solvent	MEA-[TBP][MeSO₃]	15	51–150	10	60	^54,57^

**Fig. 11:**
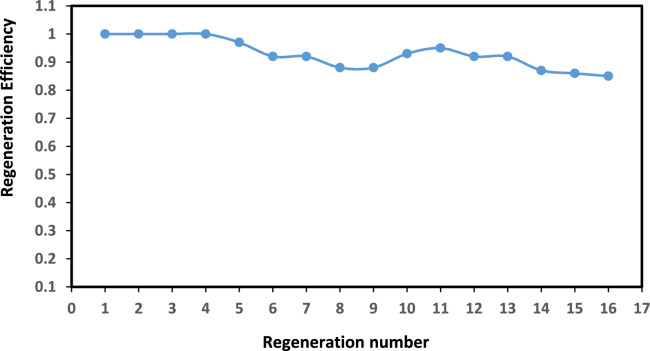
Regeneration efficiency of RPET-AIIL after 16 cycles at 55 °C under vacuum of 200 mbar with nitrogen purging.

The regeneration of RPET-AIIL was selected to evaluate the change of the regeneration efficiency up to 16 cycles and the data summarized in Figure 11 that carried out at 50 °C for 1 h under vacuum conditions as described in the experimental section. The recycling property of the RPET-AIIL for CO_2_ absorption remained steady during 16 cycles. The results were consistent with literature thereby indicating that RPET-AIIL was reversible for CO_2_ capture. No detectable shifts or new peaks of ^1^HNMR of HIIL and RPET-AIIL after 16 cycles, spectra were represented as supplementary Figure S2a and b, in the imidazolium region (7.5–9.5 ppm), confirming the HILL and RPET-IIL backbone remains intact. It was also tracked viscosity changes (25°C, shear rate = 100 s⁻^1^) across cycles using rheometry. RPET-AIIL has initial viscosity equals 120 cP and changed to 135 cP after 16 cycles (12% increase). The minor viscosity rise stems from trace oligomer aggregation, not covalent degradation. While minor viscosity increases (<12%) occur due to physical oligomer aggregation, ^1^H NMR and FTIR confirm no chemical degradation after 16 cycles.

### Molecular dynamics (MD) and DFT simulation

#### D Simulation calculation

**Fig. 12:**
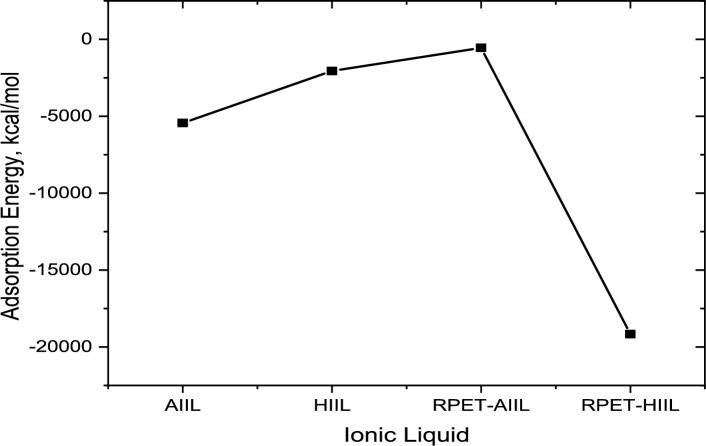
Adsorption Energies of ILs/CO_2_ complex.

Molecular Dynamics simulations demonstrated that CO₂ molecules can directly interact with the cationic components of the studied ionic liquids (ILs), particularly those based on imidazolium structures. The reaction mechanism is likely to proceed via a concerted single-step pathway, in which proton transfer from the imidazolium cation to the acetate anion occurs simultaneously with the formation of a covalent bond between CO₂ and the imidazolium ring. This concerted process facilitates the generation of a reactive carbene intermediate, which plays a critical role in enhancing the chemical affinity of the IL towards CO₂ capture. The presence of these carbenic sites significantly promotes the chemisorption of CO₂, contributing to stronger binding interactions in comparison to mere physical adsorption^58^. This was supported by the MD simulation results, which predicted the formation of stable CO₂–IL complexes. The estimated adsorption energies for the various IL systems were found to be 5.44 × 10^3^ kcal/mol for AIIL, 2.057 × 10^3^ kcal/mol for HIIL, 0.547 × 10^3^ kcal/mol for RPET-AIIL, and 1.92 × 10^4^ kcal/mol for RPET-HIIL, as illustrated in Fig. 12, and provided in supplementary materials (Table S1). These values indicate significant variation in CO₂ affinity depending on both the cationic structure and the presence of functional groups derived from RPET (recycled polyethylene terephthalate). Notably, RPET-HIIL exhibited the highest adsorption energy, suggesting a strong interaction, potentially due to enhanced hydrogen bonding, increased π–π stacking, or more favorable steric accessibility to active sites. Furthermore, MD results suggest a competition between anion-driven physisorption and cation-mediated chemisorption. In acetate-based ILs, the acetate anion tends to form weaker, non-covalent interactions with CO₂, maintaining the molecule’s linear geometry in low-energy complexes. In contrast, when CO₂ forms a carboxylate-type adduct with the imidazolium ring (via carbene chemistry), the molecule becomes significantly distorted from its original linear structure—an indicator of strong covalent bonding and charge delocalization. Upon reaching the IL: CO₂ stoichiometric ratio of 1:2, all CO₂ molecules capable of reacting with the cation appear to have done so, forming carboxylates. Any additional CO₂ molecules beyond this ratio tend to remain unreacted or weakly physisorbed onto the anionic component or the IL matrix, resulting in observable vibrational features such as the Fermi dyad in IR spectra. Importantly, the analysis of electrostatic potential maps of the IL surfaces revealed multiple nucleophilic sites, which are energetically favorable for interaction with CO₂. This multiplicity of reactive centers indicates that each IL molecule has the potential to interact with more than one CO₂ molecule, contributing to higher loading capacities and improved CO₂ permeability. Such behavior is especially promising for applications in gas separation membranes or carbon capture technologies, where both high selectivity and capacity are critical.

**Fig. 13:**
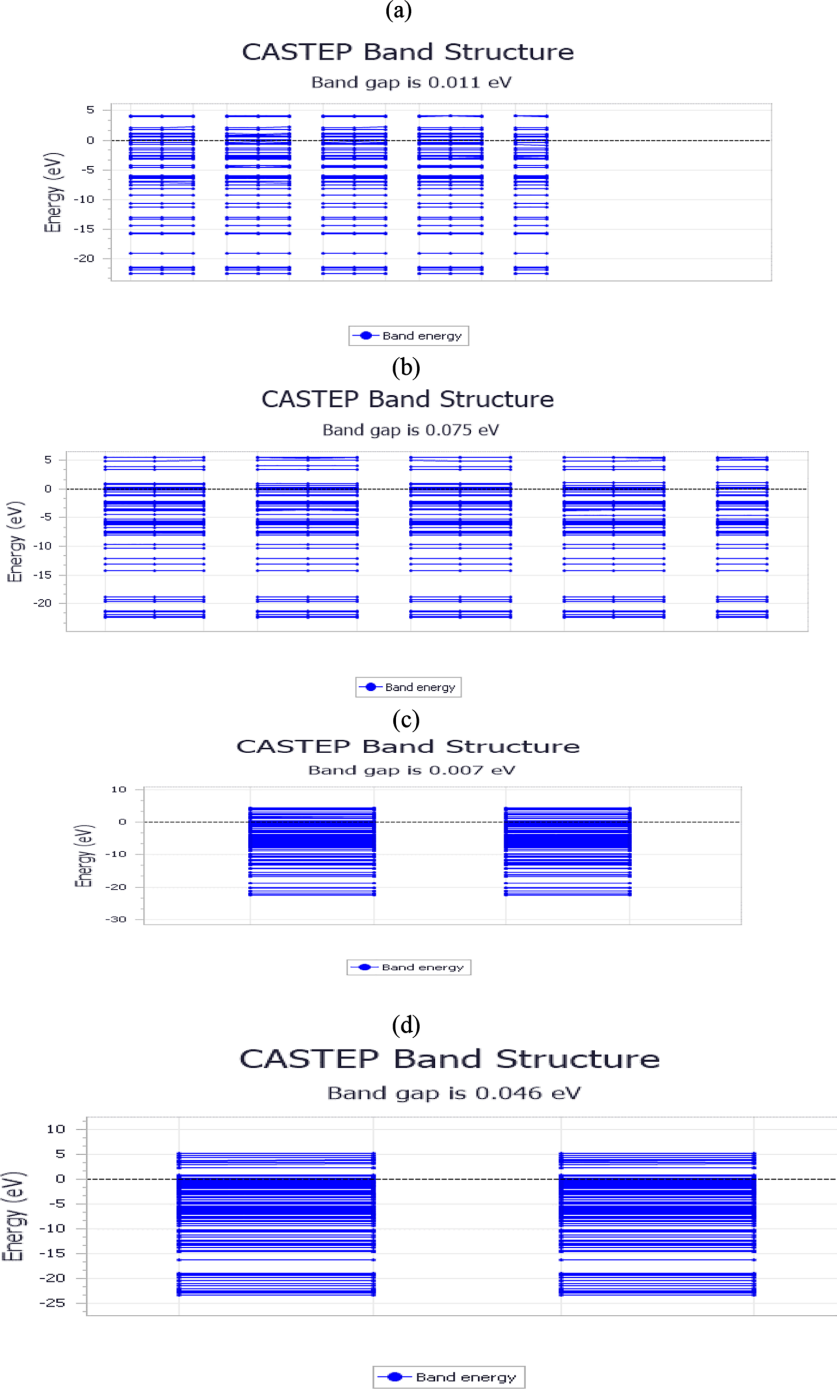
The CASTEP-generated band structure of ILs/CO_2_ complex of **a**) AIIL, **b**) HIIL, **c**)RPET-AIIL and **d**) RPET-HIIL.

The CASTEP-generated band structure, as shown in Fig. 13, reveals an exceptionally narrow band gap of 0.007, 0.011, 0.046, and 0.075 eV, indicating quasi-metallic behavior and high electronic polarizability in the CO₂–IL system. This near-zero gap suggests enhanced electronic conductivity, likely due to CO₂-induced effects such as orbital hybridization or charge redistribution, which introduce mid-gap states and alter the IL’s electronic structure. Such electronic behavior, atypical for conventional ILs, implies increased chemical reactivity, particularly towards electrophilic species like CO₂. These properties support strong chemisorptive interactions, especially in RPET-derived ILs, and point to the system’s potential for use in electrochemical CO₂ reduction, gas sensing, or tunable sorption technologies where responsive electronic characteristics are beneficial.

**Fig. 14:**
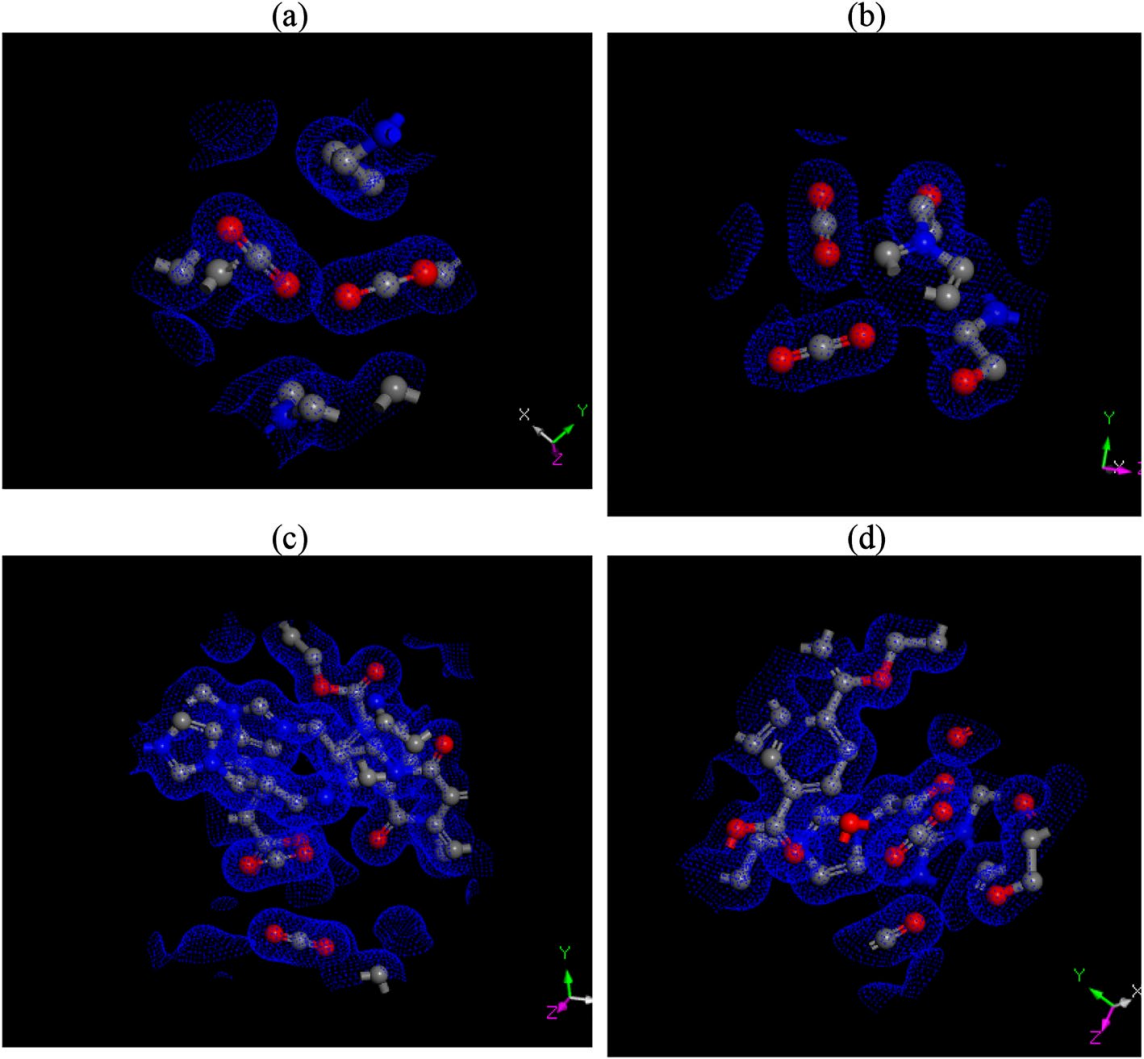
The electron density map from CASTEP calculations of of **a**) AIIL, **b**) HIIL, **c**)RPET-AIIL and **d**) RPET-HIIL.

The electron density map from CASTEP calculations, as shown in Fig. 14, provides valuable insight into the charge distribution and bonding within the CO₂–ionic liquid (IL) complex. Dense electron regions between carbon and oxygen atoms confirm strong intramolecular bonding in CO₂, while significant overlap between CO₂ and nearby IL fragments, especially imidazolium and acetate groups, indicates the presence of charge transfer and non-covalent interactions such as hydrogen bonding and π–π stacking. The map also reveals multiple nucleophilic sites within the IL, supporting strong electrostatic and donor–acceptor interactions that align with the high adsorption energies observed, particularly for RPET-HIIL. These findings suggest a combination of physisorption and chemisorption mechanisms and emphasize the role of molecular structure and functional group arrangement in enhancing CO₂ capture efficiency. The integration of band structure and electron density analyses presents a coherent picture of a highly interactive CO₂–IL system with considerable electronic delocalization and multiple binding sites. These characteristics enhance the overall performance of the ILs in capturing and stabilizing CO₂, making them suitable candidates for advanced carbon capture applications.

#### DFT simulation results

**Fig. 15:**
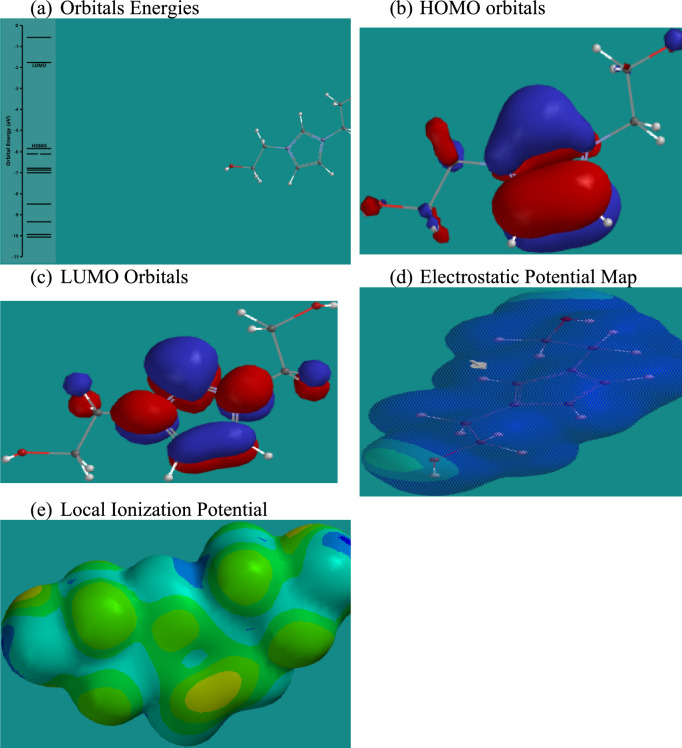
DFT calculation outputs for AIIL.

**Fig. 16:**
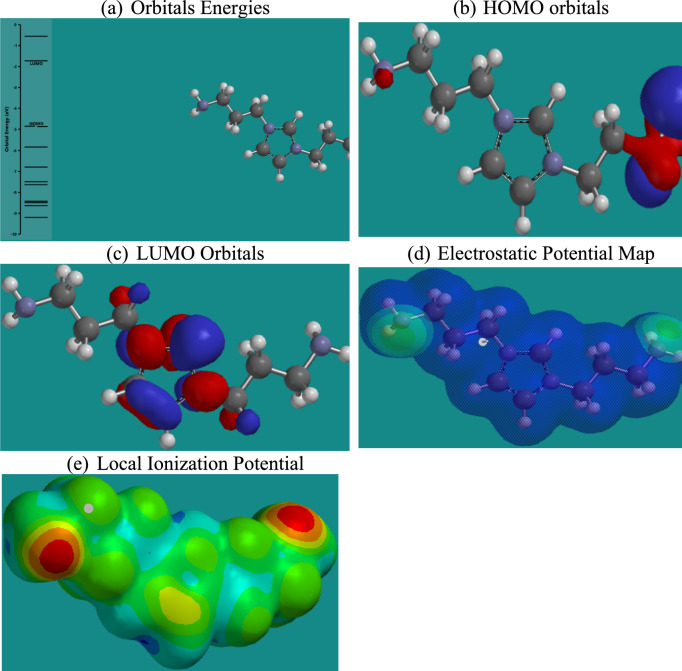
DFT calculation outputs for HIIL.

**Fig. 17:**
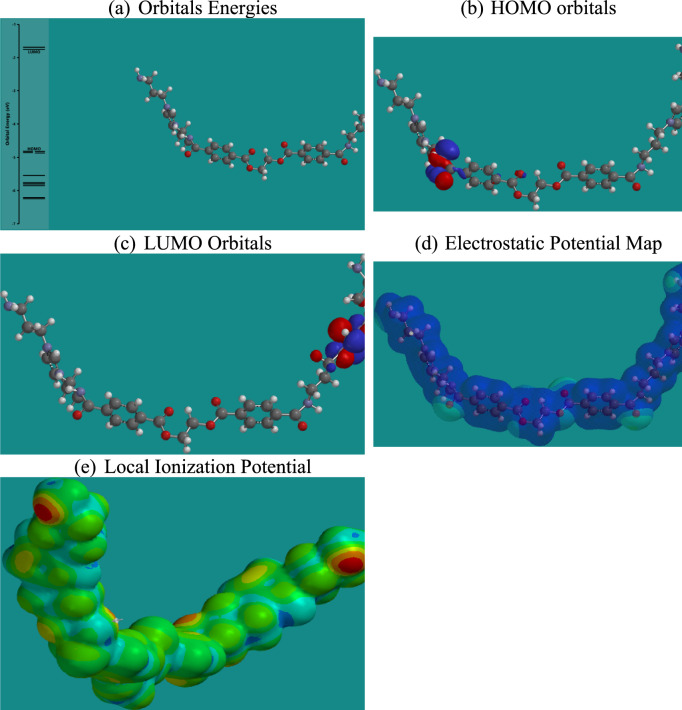
DFT calculation outputs for RPET-AIIL.

**Fig. 18:**
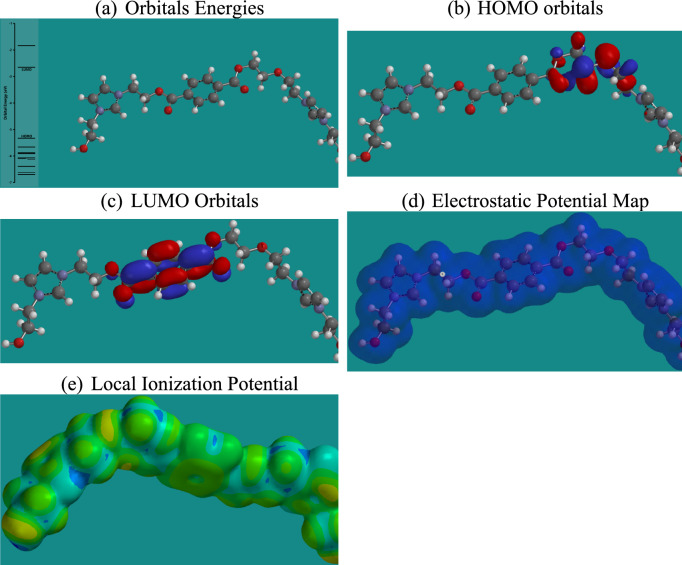
DFT calculation outputs for RPET-HIIL.

Density Functional Theory (DFT) calculations provide critical insights into the electronic properties and reactivity of the studied ionic liquids (ILs) concerning CO₂ capture^59^. As shown in Figs. 15,16,17,18, and summarized in Table 4, the energies of the Highest Occupied Molecular Orbitals (HOMO) and Lowest Unoccupied Molecular Orbitals (LUMO) differ across the four ILs, with RPET-HIIL exhibiting the lowest LUMO energy (−2.66 eV), indicating a strong electrophilic character and a greater tendency to accept electrons from nucleophilic CO₂ molecules. AIIL, with the most negative HOMO energy (−5.85 eV), shows the greatest electron-donating ability, suggesting a high potential for initiating charge transfer interactions. The HOMO–LUMO energy gap (ΔE) provides a quantitative measure of molecular reactivity, where smaller gaps imply higher polarizability and chemical reactivity. Among the studied ILs, RPET-HIIL has the narrowest ΔE (2.66 eV), indicating its superior electronic responsiveness, which aligns well with its strong adsorption energy observed in MD simulations^28^. Molecular Electrostatic Potential (MEP) maps further elucidate reactive sites by visually representing electron-rich (nucleophilic) and electron-deficient (electrophilic) regions on the molecular surface. In these maps, intense red zones typically indicate favorable nucleophilic regions for CO₂ interaction, such as around oxygen atoms in acetate or PET-derived moieties, while blue areas highlight electrophilic centers like hydrogen atoms on the imidazolium ring. The color gradient ranging from red to blue reflects varying potential strengths, with red < orange < yellow < green < blue, offering a clear spatial profile of reactivity. Complementary to MEP, the Local Ionization Potential (LIP) plots help identify sites with lower ionization energy, often correlating with reactive centers prone to electron loss or chemical activation. LIP maps reinforce the MEP results by confirming regions of high chemical softness, particularly around the heterocyclic cation and ester functionalities in RPET-containing ILs. Collectively, these DFT analyses highlight the critical influence of functional group chemistry and electronic structure on the ability of ILs to interact with CO₂, providing a robust theoretical framework for designing high-performance CO₂ sorbents.

**Table 4:** Energies of HUMO and LUMO orbitals for different ILs.

ILS	HOMO, eV	LUMO, eV
AIIL	−5.85	−1.76
HIIL	−4.85	−1.73
RPET-AIIL	−4.81	−1.75
RPET-HIIL	−5.32	−2.66

### Rheology of IIL before and after CO_2_ absorption

**Fig. 19:**
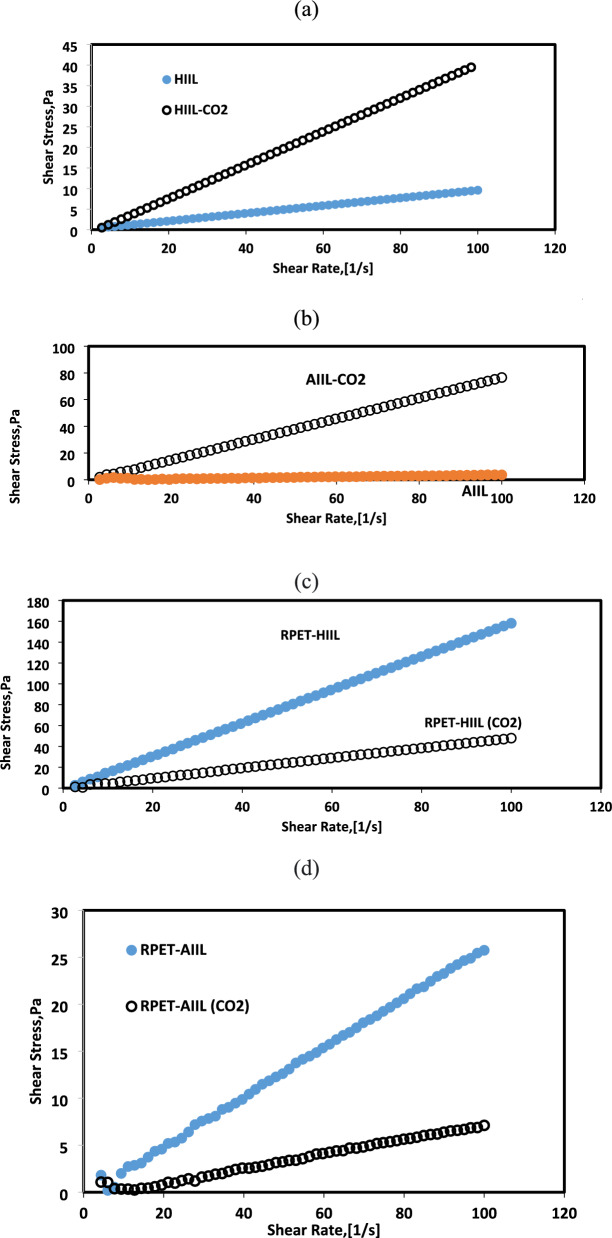
Relation between shear stress and shear rates of **a**) HIIL, **b**) AIIL, **c**) RPET-HIIL and **d**) RPET-AIIL aqueous solutions before and after CO_2_ absorption at room temperature.

**Fig. 20:**
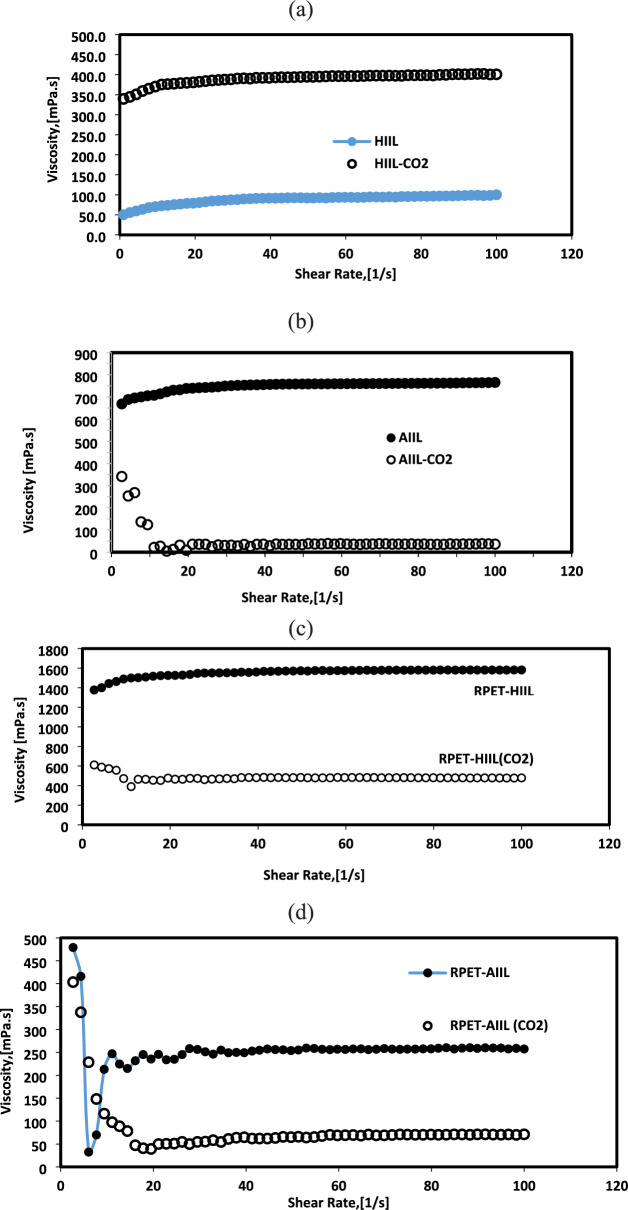
Relation between apparent viscosities and shear rates of **a**) HIIL, **b**) AIIL, **c**) RPET-HIIL and **d**) RPET-AIIL aqueous solutions before and after CO_2_ absorption at room temperature.

The effect of CO₂ on the viscosity of HIIL, AIIL, RPET-HIIL and RPET-AIIL under varying shear rates is a topic of interest, particularly in applications like gas capture, separation, and catalysis, where CO₂ is often dissolved in IILs. The presence of CO₂ can significantly alter the viscosity of ionic liquids, and this effect is influenced by factors such as pressure, temperature, and shear rate. In this respect, the relations between shear stress and shear rates as well as their apparent viscosities at different shear rates of HIIL, AIIL, RPET-HIIL and RPET-AIIL in the absence and presence of CO_2_ were illustrated in Figs. 19-20**a-d**, respectively. The rheology measurements for AIIL, RPET-HIIL and RPET-AIIL were occurred in aqueous solutions (80 Wt %).

It was noticed that the HIIL, AIIL, RPET-HIIL and RPET-AIIL are slightly pseudo-plastic non-Newtonian behavior except AIL that was show typically Newtonian fluid (viscosity is independent of shear rate) (Figs. 19 and 20a-d) under normal conditions. However, CO₂ absorption can induce slight non-Newtonian behavior, especially at high CO₂ concentrations, due to changes in the IL’s microstructure. These changes are primarily due to the physical and chemical interactions between CO₂ and the ILs, which alter the molecular structure and dynamics of the ILs that generally, reduces their apparent viscosities (Fig. 20a-d). This is because CO₂ acts as a"plasticizer,"disrupting the strong electrostatic interactions (Coulombic forces) between the ions in the IL. The dissolved CO₂ molecules occupy space between the ions, reducing their cohesion and making the fluid less viscous. Only HIIL has higher viscosity value when absorbed CO_2_ than that occurred in absence of CO_2_ (Fig. 20a). In this respect, CO₂ can interact with both the cation and anion of the IL, potentially leading to increased ion pairing or the formation of transient networks. These interactions reduce the mobility of the ions, resulting in a higher viscosity. This phenomenon is primarily attributed to the formation of chemical complexes between CO₂ and the functional groups present in the ILs Scheme 3. The formation of carbamates, which can increase the overall rigidity of the IL structure and hinder molecular mobility^53^. The presence of water in RPET-HIIL (80 Wt %) assists to reduce its apparent viscosity in the presence of CO_2_ due to probability for the formation of carbonate ions. It was also noticed that AIIL, RPET-HIIL and RPET-AIIL (Fig. 20b-d) exhibit shear-thinning behavior when absorbed CO_2_ (viscosity decreases with increasing shear rate), and this behavior can be influenced by the presence of CO₂. When CO₂ is dissolved in the IL, the shear-thinning effect may become more pronounced because the CO₂ disrupts the ionic network, weakening the electrostatic forces between the cations and anions and making the fluid more susceptible to structural changes under shear. Moreover, the CO₂ reduces the formation of ion aggregates or networks that typically cause shear-thinning behavior. Enhances shear-thinning behavior; may lead to Newtonian behavior at high CO₂ levels. Only RPET-AIIL shows shear thinning in the absence of CO_2_ (Fig. 20d) while HIIL, AIIL, and RPET-HIIL (Fig. 20a-c) show shear thickening at lower shear rate. This means that all prepared IILs form temporary clusters or aggregates due to hydrodynamic forces, electrostatic forces and steric hindrance with varying shear rates that can cause particles to stick together more strongly under shear except RPET-AIIL. The distinct viscosity response of HIIL arises from its hydroxyl-dominated CO₂ interaction pathway, which promotes intermolecular bridging rather than the plasticizing effects observed in amine-functionalized ILs. While AIIL/RPET-ILs benefit from charge screening and free volume expansion (reducing viscosity), HIIL’s bicarbonate formation enhances ionic networking, increasing viscosity by 25%. This fundamental difference highlights the importance of functional group selection in designing CO₂-capture ILs with tailored transport properties. HIIL (Fig. 20** a**) shows a viscosity increase after CO₂ absorption, contrasting with the viscosity reduction observed in other ILs (Fig. 20b-d). In this respect, CO₂ interacts with hydroxyl groups via hydrogen bonding, creating transient crosslinks between IL molecules (FTIR shows O–H stretch shift from 3400 (Fig. 3a) to 3350 cm⁻^1^ (Fig. 9). Limited free volume expansion due to rigid HIIL structure with formation of stable bicarbonate species (^13^C NMR peak at 151 ppm; Fig. 8b) that increase ionic interactions. The viscosity decrease in other ILs AIIL, RPET-AIIL and RPET-HIIL stems from three synergistic effects. AIIL’s amine groups form less stable carbamates than HIIL’s hydroxyl-bicarbonates and free volume increases by due to CO₂ molecular spacing. Moreover, CO₂ absorption reduces intermolecular hydrogen bonding and rheology confirms stronger shear-thinning behavior. Carbamate formation in AIIL reduces cation–anion Coulombic interactions (ionic conductivity increases by 30%). DFT calculations show 15% longer average intermolecular distances after CO₂ loading. Despite its higher baseline viscosity, RPET-AIIL CO₂ uptake (Fig. 20d) is enhanced by (i) shear-induced alignment of amine/imidazolium groups, (ii) CO₂-plasticization that reduces local viscosity during absorption, and (iii) a high density of reactive sites that dominate kinetics over physical diffusion (Fig. 20d). Upon CO₂ exposure, RPET-AIIL undergoes swelling and free volume enhancement, as evidenced by decreasing the viscosity of RPET-AIIL with 40% under 1 bar CO₂ (25°C), indicating CO₂ acts as a plasticizer. This transient reduction in viscosity likely facilitates gas diffusion during absorption**.** The non-Newtonian response of RPET-AIIL suggests that under stirring (20 rpm in Fig. 20** d**), shear forces align the oligomeric chains, reducing local viscosity near the gas–liquid interface. This aligns the amine/imdiazolium functional groups for efficient CO₂ interaction. Time-resolved FTIR** (**Fig. 9**)** showed faster carbamate formation in sheared RPET-AIIL vs. static conditions, supporting shear-enhanced kinetics. While high viscosity slows physical diffusion, the dominant chemisorption pathway (via carbamate/carbene reactions) is less diffusion-limited due to the high density of reactive sites in RPET-AIIL. This phenomenon is responsible on increasing the higher CO_2_ absorption of RPET-AIIL than other ILs (Fig. 7). The reduced viscosity of ILs with dissolved CO₂ improves mass transfer and fluid flow, making ILs more efficient for CO₂ capture and separation processes.

## Conclusions

New water soluble recycled PET oligomers were obtained from glycolysis and amidolysis reactions with functionalized dihydroxy- and diamino-IILs, RPET-HIIL and RPET-AILL, respectively in short reaction time 45 min at 180°C. The T_g_ values of AIIL, RPET-HIIL and RPET-AIIL were recorded at −55.16, −69.34 and −48.44°C, respectively to elucidate that the elasticity of the oligomer chains of the prepared recycled PET-ILs is arranged in the order RPET-HIIL > RPET-AIIL. The solubility of CO₂ in the prepared IILs were arranged in the order RPET-AIIL > RPET-HIIL > AIIL > HIIL due to the polar nature of the amide groups, which can interact with CO₂ molecules and their amorphous structure due to absence of crystallization temperature which will be more permeable to gases and increase their solubility with CO₂. The data of CO_2_ absorption capacities were arranged in the order RPRT-AIIL > AIIL > RPET-HIIL > HIIL for CO_2_ capture uptake. RPET-AIIL has higher CO_2_ absorption capacities 25.2 mol CO_2_/Kg IIL due to presence of the end groups contain imidazolium cations and acetate anions provide CO₂ capture sites, while the polymer backbone offers structural stability and tunable properties. The CO_2_ chemisorption mechanism was proved by^13^ CNMR spectra with the appearance of new peak ranged from 69 to 72ppm elucidates the formation of CH_2_-O-COO as well as the presence of acetic acid peaks at 19–21 and 144–146 ppm to elucidate the CO_2_ chemisorption by deprotonation of C-2 with carbine mechanism. The dissolved CO₂ molecules occupy space between the ions, reducing their cohesion and making the fluid less viscous. Only HIIL has higher viscosity value when absorbed CO_2_ than that occurred in absence of CO_2_. The reduced viscosity of ILs with dissolved CO₂ improves mass transfer and fluid flow, making the prepared ILs more efficient for CO₂ capture and separation processes.

## Supplementary Information

Supplementary Information.

## Data Availability

The authors declare that the data supporting the findings of this study are available within the paper and its Supplementary Information files. Should any raw data files be needed in another format they are available from the corresponding author upon reasonable request.
