# Supplementary material for: New recycled polyethylene terephthalate imidazolium ionic liquids and their applications for co2 exhaust capture
Source: Sci Rep. 2025 Aug 19;15:30378. doi: 10.1038/s41598-025-15433-7 (PMC12365022; doi:10.1038/s41598-025-15433-7)
Supplement: Supplementary file 1 — Supplementary Information. [file 41598_2025_15433_MOESM1_ESM.docx]

**New Recycled Polyethylene Terephthalate Imidazolium Ionic Liquids and Their Applications for CO_2_ Exhaust Capture**

**Ayman M. Atta^1*^, Alia A. Melegy^1^, A.N. El-hoshoudy^2,3^**

^1^Petroleum Application department, Egyptian Petroleum Research Institute, Nasr City 11727, Cairo, Egypt.

^2^PVT Lab, Production Department, Egyptian Petroleum Research Institute, Nasr City 11727, Cairo, Egypt.

^3^PVT Service Center, Egyptian Petroleum Research Institute, Nasr City 11727, Cairo, Egypt.

Corresponding authors: **Ayman M. Atta; khaled_00atta@yahoo.com**

Table S1: Adsorption Energies of ILs/CO_2_

| Structures | Total energy | Adsorption energy | Rigid adsorption energy | Deformation energy | HIIL : dEad/dNi | CO2 : dEad/dNi |
| --- | --- | --- | --- | --- | --- | --- |
| AIIL - 1 | 31.53269716 | -2.06E+03 | -7.36032926 | -2.05E+03 | -1.86E+03 | -103.1797277 |
| AIIL - 2 | 32.07512541 | -2.06E+03 | -6.82011135 | -2.05E+03 | -1.85E+03 | -102.657166 |
| AIIL - 3 | 32.82352488 | -2.06E+03 | -6.07547616 | -2.05E+03 | -1.85E+03 | -102.4281446 |
| AIIL - 4 | 33.24784122 | -2.06E+03 | -7.61813379 | -2.05E+03 | -1.85E+03 | -103.242376 |
| AIIL - 5 | 33.25160909 | -2.06E+03 | -7.61877627 | -2.05E+03 | -1.85E+03 | -103.3774771 |
| AIIL - 6 | 33.6320011 | -2.06E+03 | -7.23380987 | -2.05E+03 | -1.85E+03 | -102.8357433 |
| AIIL - 7 | 33.52539433 | -2.06E+03 | -7.34001476 | -2.05E+03 | -1.85E+03 | -103.7253802 |
| AIIL - 8 | 33.71048768 | -2.06E+03 | -7.15519289 | -2.05E+03 | -1.85E+03 | -102.6178352 |
| AIIL - 9 | 33.94979266 | -2.06E+03 | -6.91557504 | -2.05E+03 | -1.85E+03 | -102.56748 |
| AIIL - 10 | 34.23342769 | -2.06E+03 | -4.6775206 | -2.05E+03 | -1.85E+03 | -101.3143599 |
|  |  |  |  |  |  |  |
| Average | 33.19819012 | -2058.1789 | -6.881493999 | -2051.2974 | -1854.0837 | -102.794569 |
|  |  |  |  |  |  |  |
| Structures | Total energy | Adsorption energy | Rigid adsorption energy | Deformation energy | HIIL (2) : dEad/dNi | CO2 : dEad/dNi |
| HIIL - 1 | 32.67333735 | -2.06E+03 | -8.18942878 | -2.05E+03 | -1.85E+03 | -103.3328679 |
| HIIL - 2 | 33.0170104 | -2.06E+03 | -7.85251371 | -2.05E+03 | -1.85E+03 | -102.9853549 |
| HIIL - 3 | 32.99824772 | -2.06E+03 | -7.86636297 | -2.05E+03 | -1.85E+03 | -103.1758003 |
| HIIL - 4 | 33.32537686 | -2.06E+03 | -7.53893981 | -2.05E+03 | -1.85E+03 | -102.7839464 |
| HIIL - 5 | 34.61935288 | -2.06E+03 | -6.24444601 | -2.05E+03 | -1.85E+03 | -101.3143464 |
| HIIL - 6 | 34.66082962 | -2.06E+03 | -6.20291271 | -2.05E+03 | -1.85E+03 | -101.3143822 |
| HIIL - 7 | 34.51354703 | -2.06E+03 | -6.35458554 | -2.05E+03 | -1.85E+03 | -101.3143818 |
| HIIL - 8 | 34.95006407 | -2.06E+03 | -5.92823318 | -2.05E+03 | -1.85E+03 | -101.314384 |
| HIIL - 9 | 35.60494912 | -2.06E+03 | -5.26183465 | -2.05E+03 | -1.85E+03 | -101.314357 |
| HIIL - 10 | 35.73927471 | -2.06E+03 | -5.12398742 | -2.05E+03 | -1.85E+03 | -101.8720775 |
|  |  |  |  |  |  |  |
| Average | 34.21019898 | -2057.167 | -6.656324478 | -2050.5105 | -1853.4596 | -102.0721898 |
|  |  |  |  |  |  |  |
| Structures | Total energy | Adsorption energy | Rigid adsorption energy | Deformation energy | RPET-AIIL (2) : dEad/dNi | CO2 : dEad/dNi |
| RPET-AIIL - 1 | 82.31511378 | -550.2580053 | -34.09225773 | -516.1657476 | -345.9763714 | -101.3143226 |
| RPET-AIIL - 2 | 82.55627747 | -550.0168416 | -33.82342817 | -516.1934135 | -345.6815662 | -101.314352 |
| RPET-AIIL - 3 | 83.20012947 | -549.3729896 | -33.59610174 | -515.7768879 | -345.0255165 | -101.3143288 |
| RPET-AIIL - 4 | 83.40687036 | -549.1662487 | -32.99501897 | -516.1712298 | -346.41934 | -101.3143739 |
| RPET-AIIL - 5 | 83.68792956 | -548.8851895 | -32.70785355 | -516.177336 | -346.1391999 | -101.3143538 |
| RPET-AIIL - 6 | 85.56242014 | -547.010699 | -30.83859547 | -516.1721035 | -344.3819933 | -101.3143108 |
| RPET-AIIL - 7 | 86.09304719 | -546.4800719 | -30.54431126 | -515.9357607 | -343.851394 | -101.3143366 |
| RPET-AIIL - 8 | 87.05279704 | -545.5203221 | -31.32547269 | -514.1948494 | -342.8915931 | -101.31436 |
| RPET-AIIL - 9 | 87.35497037 | -545.2181487 | -31.16136269 | -514.0567861 | -342.5911656 | -101.312792 |
| RPET-AIIL - 10 | 87.80766747 | -544.7654516 | -28.87054512 | -515.8949065 | -342.1367429 | -101.3143541 |
|  |  |  |  |  |  |  |
| Average | 84.90372229 | -547.6693968 | -31.99549474 | -515.6739021 | -344.5094883 | -101.3141884 |
| Structures | Total energy | Adsorption energy | Rigid adsorption energy | Deformation energy | RPET-HIIL (2) : dEad/dNi | CO2 : dEad/dNi |
| RPET-HIIL - 1 | 79.61607463 | -1.92E+04 | -15.14368089 | -1.92E+04 | -1.90E+04 | -103.7177901 |
| RPET-HIIL - 2 | 81.32371004 | -1.92E+04 | -13.76984372 | -1.92E+04 | -1.90E+04 | -102.1739601 |
| RPET-HIIL - 3 | 81.94774495 | -1.92E+04 | -14.95349787 | -1.92E+04 | -1.90E+04 | -103.0365829 |
| RPET-HIIL - 4 | 82.1780542 | -1.92E+04 | -14.55747427 | -1.92E+04 | -1.90E+04 | -102.1501666 |
| RPET-HIIL - 5 | 82.4414981 | -1.92E+04 | -12.54649787 | -1.92E+04 | -1.90E+04 | -101.3143523 |
| RPET-HIIL - 6 | 82.92946711 | -1.92E+04 | -13.95711138 | -1.92E+04 | -1.90E+04 | -102.4736881 |
| RPET-HIIL - 7 | 83.22157252 | -1.92E+04 | -14.44529169 | -1.92E+04 | -1.90E+04 | -102.21065 |
| RPET-HIIL - 8 | 83.43127459 | -1.92E+04 | -14.24704359 | -1.92E+04 | -1.90E+04 | -102.2385368 |
| RPET-HIIL - 9 | 84.39485198 | -1.92E+04 | -11.06578644 | -1.92E+04 | -1.90E+04 | -101.3143478 |
| RPET-HIIL - 10 | 84.65331702 | -1.92E+04 | -10.57458016 | -1.92E+04 | -1.90E+04 | -101.3143586 |
|  |  |  |  |  |  |  |
| Average | 82.61375651 | -19166.38 | -13.52608079 | -19152.855 | -18962.89 | -102.1944433 |

| 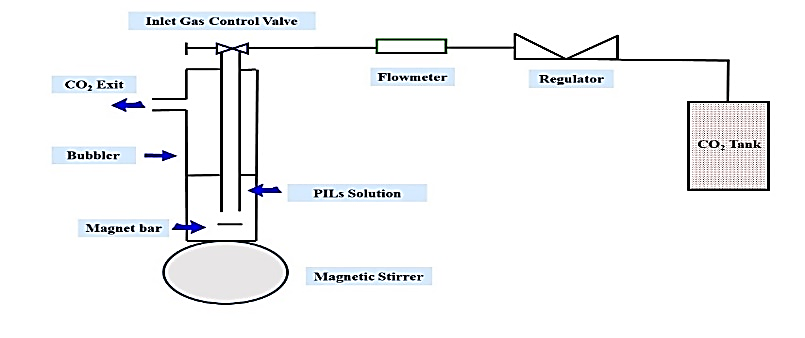 |
| --- |
| Figure S1: Schematic representation of bubbler gas system for CO_2_/IILs.  (a)    (b)   |

Figure S2. ^1^ HNMR spectra of a) HIIL and b) RPET-AIIL after 10 regenerated cycles.
